# Rewiring of Metabolic Network in *Mycobacterium tuberculosis* During Adaptation to Different Stresses

**DOI:** 10.3389/fmicb.2019.02417

**Published:** 2019-10-29

**Authors:** Arshad Rizvi, Arvind Shankar, Ankita Chatterjee, Tushar H. More, Tungadri Bose, Anirban Dutta, Kannan Balakrishnan, Lavanya Madugulla, Srikanth Rapole, Sharmila S. Mande, Sharmistha Banerjee, Shekhar C. Mande

**Affiliations:** ^1^Department of Biochemistry, School of Life Sciences, University of Hyderabad, Hyderabad, India; ^2^Bio-Sciences R&D Division, TCS Research, Tata Consultancy Services Ltd., Pune, India; ^3^National Centre for Cell Science, Pune, India

**Keywords:** genome-scale metabolic network, metabolite profiling, metabolic rewiring, stress adaptation in *M. tuberculosis*, sugar alcohols

## Abstract

Metabolic adaptation of *Mycobacterium tuberculosis* (*M. tuberculosis*) to microbicidal intracellular environment of host macrophages is fundamental to its pathogenicity. However, an in-depth understanding of metabolic adjustments through key reaction pathways and networks is limited. To understand how such changes occur, we measured the cellular metabolome of *M. tuberculosis* subjected to four microbicidal stresses using liquid chromatography-mass spectrometric multiple reactions monitoring (LC-MRM/MS). Overall, 87 metabolites were identified. The metabolites best describing the separation between stresses were identified through multivariate analysis. The coupling of the metabolite measurements with existing genome-scale metabolic model, and using constraint-based simulation led to several new concepts and unreported observations in *M. tuberculosis*; such as (i) the high levels of released ammonia as an adaptive response to acidic stress was due to increased flux through L-asparaginase rather than urease activity; (ii) nutrient starvation-induced anaplerotic pathway for generation of TCA intermediates from phosphoenolpyruvate using phosphoenolpyruvate kinase; (iii) quenching of protons through GABA shunt pathway or sugar alcohols as possible mechanisms of early adaptation to acidic and oxidative stresses; and (iv) usage of alternate cofactors by the same enzyme as a possible mechanism of rewiring metabolic pathways to overcome stresses. Besides providing new leads and important nodes that can be used for designing intervention strategies, the study advocates the strength of applying flux balance analyses coupled with metabolomics to get a global picture of complex metabolic adjustments.

## Introduction

*Mycobacterium tuberculosis* (*M. tuberculosis*), tuberculosis (TB) causing bacteria in human, is a very successful pathogen, that still claims millions of lives worldwide every year (World Health Organization [WHO], 2018). The success of this pathogen, in part, is attributed to its ability to survive long periods of slow or arrested growth, remaining quiescent for years before reactivating to cause active disease (Flynn and Chan, 2001). The clinical evidence of the occurrence of *M. tuberculosis* within diverse forms of lesions during different stages of infection (Lenaerts et al., 2015) strongly indicates its competence to survive a variety of permissive and restrictive host environments. Aerosolic *M. tuberculosis* typically enters the lungs and ingested by alveolar macrophages, where it is challenged by microbicidal environment comprising of low pH, reactive oxygen and nitrogen intermediates (ROI and RNI) in concomitance with deprivation of nutrients and essential micronutrients (Denis, 1991; Schaible et al., 1998; Gomez and McKinney, 2004).

Studies involving genomics, transcriptomics, and proteomics have unraveled a wide array of molecular mechanisms employed by *M. tuberculosis* to survive this hostile microbicidal milieu of macrophage phagosomes (Forrellad et al., 2013; Gopinath et al., 2015; Liu et al., 2016; Danelishvili et al., 2017; Hoffmann et al., 2018). This included mycobacterial detoxification system of catalase (KatG) (Ng et al., 2004), superoxide dismutases (SodA and SodC) (Wu et al., 1998; Dussurget et al., 2001; Piddington et al., 2001; Sassetti and Rubin, 2003), mycothiol (MSH) (Buchmeier and Fahey, 2006; Buchmeier et al., 2006; Vilcheze et al., 2008), NADH-dependent peroxidase, and peroxynitrite reductase system (Bryk et al., 2000, 2002; Tian et al., 2005a; Shi and Ehrt, 2006). Studies also advocate the role of cell wall components like OmpA (Outer membrane protein A) in resisting low pH, with mutant screening appropriately indicating the crucial role of biosynthesis of peptidoglycan or the cell wall lipid lipoarabinomannan in intracellular survival (Raynaud et al., 2002; Sassetti and Rubin, 2003; Molle et al., 2006; Vandal et al., 2009). Proton pumps and unconventional transporters, like Mg^2+^ transporter MgtC, have also been suggested to be important for intra-phagosomal survival of *M. tuberculosis*. Besides these, there are reports on pathways involving DNA and protein repair or degradation to play direct or indirect roles in the pathogenesis of *M. tuberculosis* (Forrellad et al., 2013; Ehrt et al., 2015, and references therein).

While these studies point to an array of essential proteins and pathways that may be regulated at genomic or transcript levels in *M. tuberculosis*, an early response is often met by changes in the metabolites and activities of these proteins which control the rate of turnover of molecules through metabolic networks. For instance, NADH-dependent peroxidase and peroxynitrite reductase in *M. tuberculosis* is instrumental in resisting reactive oxygen intermediated (ROI) and reactive nitrogen intermediated (RNI). The complex consists of four enzymes, alkyl hydroperoxide reductase subunit C (AhpC), a thioredoxin-related oxidoreductase (AhpD), dihydrolipoamide acyltransferase (DlaT) and lipoamide dehydrogenase (Lpd). DlaT and Lpd also act as E2 and E3 elements of pyruvate dehydrogenase (PDH) that produces acetyl coenzyme A (CoA) (Tian et al., 2005b), a central metabolite for many biochemical pathways. The significance of metabolic adjustments was also evident with glyoxylate shunt enzyme isocitrate lyase been proven essential for intracellular persistence of *M. tuberculosis* in both macrophages and mice, the essentiality of which was worked out recently through a combination of chemogenetic and metabolomic approaches (McKinney et al., 2000; Eoh and Rhee, 2014). Since then, several studies involving both cellular and animal models where *M. tuberculosis* recovered from macrophages *in vitro* and from mouse lungs have implicated crucial roles of central carbon metabolism, fatty acid uptake, and utilization, biosynthesis of lysine and leucine, gluconeogenesis, etc., amongst several other interconnected metabolic pathways (Rohde et al., 2007; Schubert et al., 2015; Kurthkoti et al., 2017; Zimmermann et al., 2017; Baker and Abramovitch, 2018; Koen et al., 2018; Lee et al., 2018). Integrating proteomics with metabolomics suggested that *M. tuberculosis* co-utilizes up to 33 different nutrients during macrophage infection, highlighting nutrient requirements and adjustments (Zimmermann et al., 2017). In recent times, metabolomics has been applied to understand antimicrobial mechanisms, surviving iron-deprived microenvironments of human granulomas, averting intracellular toxicity, etc. in *M. tuberculosis* (Rohde et al., 2007; Schubert et al., 2015; Kurthkoti et al., 2017; Zimmermann et al., 2017; Baker and Abramovitch, 2018; Koen et al., 2018; Lee et al., 2018). However, given the metabolic overlap between human and *M. tuberculosis* cells, it is challenging to segregate and measure the metabolites from the host and the pathogen. Therefore, such metabolic studies are alternatively performed *in vitro* while mimicking the *in vivo* growth conditions.

Another concern is that only limited numbers of metabolites can be identified even when the most advanced targeted-metabolomic techniques are used. Consequently, while the measurable fractions provide valuable information regarding the metabolic state of a cell/organism, comprehending cellular metabolism in its entirety remains elusive. To this end, *in silico* techniques may be put to use to decipher cellular metabolism from data on a subset of metabolites. Genome-scale metabolic models (GEMs), which enable the integration of various omics data, can be used along with flux balance analysis (FBA) techniques to simulate perturbations in cellular metabolism (Yizhak et al., 2010; Kim and Lun, 2014; Garay et al., 2015; Lu et al., 2018). Some previous *in silico* studies have already reported changes in *M. tuberculosis* metabolism under varying growth conditions by integrating transcriptomic data into FBA simulations (Yizhak et al., 2010; Blazier and Papin, 2012; Kim and Lun, 2014; Lu et al., 2018). Such an integrative approach showed that metabolic adjustments through carbon re-routing from energy and biosynthetic precursors generating metabolic pathways to pathways for storage compound synthesis indicate a switch from active growth to dormancy (Shi et al., 2010). However, no previous genome-scale FBA simulation studies on *M. tuberculosis* have incorporated metabolomic data. A probable reason for this could be the unavailability of an appropriate algorithmic-framework/software-tool wherein metabolite concentrations measured from the targeted analysis could be directly overlayed on FBA simulations.

This study aimed to understand the early metabolic adaptation to the microbicidal stresses namely, acidic, oxidative, iron deprivation and nutrient starvation that *M. tuberculosis* undergoes upon ingestion by alveolar macrophages, using established *in vitro* growth models. Targeted liquid chromatography-mass spectrometric multiple reactions monitoring (LC-MRM/MS) metabolomic was used to measure metabolites from *M. tuberculosis* H37Rv cells (henceforth referred to as *M.tb*) cultured under different stresses. To get a comprehensive picture, the metabolomics data obtained across all stresses were analyzed using statistical approaches and FBA simulations. To this end, a previously published GEM of *M.tb* (namely, iEK1011) was manually curated so that it could best mimic the observed metabolic states of *M.tb* grown under stresses. Further, given the lack of an appropriate framework, new approaches were designed for integrating metabolite data in FBA simulations.

The metabolic measurements coupled with the *in silico* simulations, indicated a complex rewiring in *M.tb* metabolic network during stresses, with nutrient deprivation causing maximum perturbations, suggesting an intriguing association of all cellular metabolites to the nutrient availability. Some of the observed metabolic changes like the release of excess ammonia during acid stress could be comprehended as adaptations of *M.tb* to the alleviation of stresses. However, unlike the general concept, the present study showed that increased flux through L-asparaginase rather than urease might be responsible for the generation of excess ammonia as an early stress response. Additionally, metabolomics data analyses point to quenching of protons through GABA shunt pathway or sugar alcohols as possible mechanisms of early adaptation to acidic and oxidative stresses in *M.tb*. Besides these new observations, our FBA analyses suggested a probable anaplerotic path for the generation of TCA intermediates from phosphoenolpyruvate using phosphoenolpyruvate kinase under nutrient stress and for the first time indicated the use of alternative cofactors to rewire metabolic pathways to overcome stresses.

## Materials and Methods

### *In vitro* Mycobacterial Cell Culture

Mycobacteria were grown following protocols described in earlier literature (Ganji et al., 2016). Primarily *M.tb* cultures were grown in 7H9 media [consisting 0.4% glycerol (v/v), 0.05% tyloxapol (v/v) and supplemented with 10% Middlebrook Oleic Albumin Dextrose Catalase (OADC) growth supplement (v/v)] at 37°C with shaking at 180 rpm till the absorbance at OD_600_ nm reached to 0.6–0.7. Cultures were then harvested, washed, and resuspended in 10 ml of respective stress media for 36 h. The study was performed by using well-established *in vitro* models mimicking stress conditions such as acidic stress (pH 5.5), oxidative stress, iron deprivation (no iron supplemented) in Sauton’s minimal medium (Vemula et al., 2016; Balakrishnan et al., 2017; Rizvi et al., 2019). For nutrient starvation stress, 1xPBS was used as described by Loebel et al. (1933) and Rizvi et al. (2019). Any possible contamination in the culture was ruled out by using ZN staining before and after stress. Each experiment was performed twice with five technical replicates. The cell viability was checked by CFU counts.

### Identification and Quantification of Metabolites From *M.tb*

#### Extraction of Intracellular Metabolites From Mycobacterial Cells

*Mycobacterium tuberculosis* cells were extracted using a modified version of an extraction protocol previously described by More et al. (2018). Briefly, post 36 h of stress, *M.tb* cells cultures were harvested and quenched in liquid nitrogen. The cell pellets were thawed on ice and treated with 1 ml of Methanol along with 0.1 mm of zirconia beads to lyse the cells for 10 cycles using mini bead beater with an interval of 1 min on ice. The supernatant was collected after centrifuging at 1000 rpm for 45 s. The supernatant was then centrifuged at 12,000 rpm for 30 min. The upper layer phase was collected in a 1.5 ml vial, and the solvent was evaporated by speed vac. The dried powder was used for further analysis.

#### LC-MRM/MS

##### Sample preparation

The dried metabolite extract from *M.tb* were dissolved in 50 μl sample buffer (6.5:2.5:1 acetonitrile: methanol: water) and used for positive ionization mode (HILIC Chromatography). For negative ionization mode, the dried metabolite extract from *M.tb* was dissolved in 50 μl ultrapure water (T3 RPLC Chromatography). For both modes, 10 μl of the sample was injected into the mass spectrometer using Shimadzu Prominence HPLC autosampler.

##### Data acquisition

Targeted metabolomic analysis was performed using multiple reaction monitoring (MRM) based approaches, as previously described (More et al., 2018). Hundred and eight metabolite standards were purchased from Sigma-Aldrich to build in-house MRM methods. For each metabolite standard, parent ion to daughter ion transitions was selected using MS/MS fragmentation. Based on the fragmentation pattern of metabolites, 108 metabolite standards were further divided into positive and negative ionization mode. For each MRM transition, collision energy (CE) and declustering potentials (DP) were optimized. The information obtained was exported to build the acquisition methods for positive and negative ionization modes. MS data were acquired using a 4000 QTRAP triple quadrupole mass spectrometer (AB SCIEX, Foster City, CA, United States) equipped with Shimadzu Prominence binary HPLC pump (Shimadzu Corporation, Japan). For positive ionization mode, the chromatographic separation was achieved using XBridge HILIC column (Waters, Milford, MA, United States) that was eluted at 700 μl/min with a 32 min linear gradient starting from 5% mobile phase A (10 mM ammonium formate with 0.1% formic acid) increasing to 60% mobile phase B (acetonitrile with 0.1% formic acid). The column was kept at 60% mobile phase B for 3 min then returned to 5% mobile phase A for equilibration. For negative ionization mode, the chromatographic separation was achieved using ATLANTIS T3 column (Waters, Milford, MA, United States) that was eluted at 500 μL/min with a 40 min linear gradient starting with 100% mobile phase A (10 mM ammonium hydroxide with 0.1% acetic acid) increasing to 98% mobile phase B (100% MeOH). The column was kept at 98% mobile phase B for 5min then returned to 100% mobile phase A for equilibration. MRM was used to acquire targeted MS data for specific metabolites in the positive and negative ion modes. Transitions, dwell time, and collision energies were set by using Analyst 1.5 software (SCIEX, Foster City, CA, United States). MS conditions were set as following, source temperature: 400°C, interface heater: ON, curtain gas: 30, declustering potential: 90, entrance and exit potential: 10, and the two ion source gases were set at 45 (arbitrary units). Analyst 1.5 software (Sciex, Foster City, CA, United States) was used to analyze LC-MRM/MS data by manual inspection of chromatograms and for the detection of the compounds. An internal standard of known concentration of d2 L-phenylalanine was used to obtained the quantitative metabolomics data. Internal standard peak areas were used to evaluate the metabolite extraction efficiency as well as to check the instrument performance over time. Analyst quantitation wizard was used for integration of peak areas. For quality measures, samples order was randomized at the time of analysis, and integration of peaks was performed in a blinded manner. Metabolites with a minimum of 30% of base peak intensity were considered for quantitation. The peak areas obtained after integration were exported in a matrix format to a spreadsheet file for univariate and multivariate analysis.

### Statistical Analysis

The data matrix file obtained from LC-MRM/MS was used as input to perform statistical analysis by using online tool MetaboAnalyst^1^ (Xia et al., 2012). The original data obtained were normalized to OD_600_ equivalent of the number of cells at the time of harvesting post stress (OD_s_) (Miguez et al., 2018) Normalization for paired comparison was performed using the following calculation [Normalized metabolite peak area for Control Experiment = (P_c_/OD_c_) X OD_s_], where P is peak area. The normalization parameters are given in Supplementary Table S11.

Each set of data was then pre-processed to achieve normal distribution (i.e., they follow a Gaussian or Normal distribution); the methods applied for each condition are mentioned in Supplementary Table S1. Such pre-processing is an essential requirement for any statistical analysis as otherwise most of the standard statistical tests become unreliable (Xia et al., 2012). Hierarchical clustering (Ward’s algorithm) was used to generate Heat map, with the dendrogram being scaled to represent the distance between each branch (distance measure: Pearson’s correlation). The color differences in the Heat map reveal the relative concentration of each metabolite across different stresses and experiment groups. Next, univariate and multivariate analysis like fold change; *t*-test, PCA, and PLS-DA were performed to identify metabolites that were differential between control and stresses. For univariate a fold change cut-off >1.5 or <0.5, *p*-value ≤ 0.05 and FDR ≤ 0.05 were considered significant (Vinaixa et al., 2012; Cruickshank-Quinn et al., 2014; More et al., 2018). Multivariate analyses such as PCA and PLS-DA were performed wherein PLS-DA is a supervised clustering method, and PLS-DA is used to generate a VIP score plot, to identify key metabolites that contribute to group segregation. VIP is defined as the weighted sum of squares of the PLS weight, which indicates the importance of the variable to the entire model. VIP ≥ 1.0 was considered to be statistically significant (Feng et al., 2016; Ma et al., 2016; Bazurto et al., 2017; Wu et al., 2018).

Further pathway analysis module of MetaboAnalyst was used to identify pathways in *M.tb* that were differentially affected during stresses. Default pathway analysis algorithm ‘Global Test’ and ‘Relative Betweenness Centrality’ were used for analysis. The metabolic pathways with Pathway impact values ≥0.1, *p*-value (*p* < 0.05) and false discovery rate (FDR) (FDR < 0.05) was considered to be affected significantly (Liew et al., 2016). Pathway analysis identifies most significantly perturbed pathways under respective stress compared to control by utilizing pathway enrichment and topology analysis. The Pathway impact is calculated from the pathway topology analysis (Xia and Wishart, 2011).

### Calculation of Adenylate Energy Charge (AEC)

AEC was computed from the levels of ATP, ADP and AMP using the following formula AEC = ([ATP]^∗^[ADP])/([ATP] + [ADP] + [AMP]) (Atkinson and Walton, 1967).

### Measurement of Urease Activity

The urease activity was measured as per manufacturer protocol by the colorimetric method at 670 nm using urease activity assay kit (ab204697, Abcam Plc., United Kingdom). Post-exposure to respective stress conditions, *M.tb* cells were washed with ice-cold 1xPBS and then resuspended in 300 μl of 1xPBS with the protease inhibitor. Cells were then lysed by bead beating, after adding 0.1 mm zirconia beads to each sample. The cell lysate was centrifuged at 10,000 rpm at 4°C for 20 min. The supernatant was filtered by a 0.22-micron filter. The protein concentration of the resultant supernatant was measured. An equal amount of protein from each sample was taken, and the volume was made up to 10 μl. To each 10 μl of the sample, 90 μl of reaction mix (urease buffer and urea) was added. The samples were homogenized and incubated at 37°C for 30 min. Then, as per manufacturer protocol, 80 μl of reagent-1 followed by 40 μl of reagent-2 were added. Samples were incubated for 30 min at room temperature). Absorbance was measured at 670 nm in a multi-well plate reader (Biotek). All the experiments were performed at least three times.

### Estimation of Ammonia Released

The ammonia released was estimated by the colorimetric method at 430 nm by using Nessler’s reagent as described (Gouzy et al., 2014) with modifications in a 96-well plate reader. In brief, after growth under respective stresses, samples were harvested at 3,500 rpm. The resultant supernatant was filtered by a 0.22-micron filter. To 10 μl of the supernatant, 90 μl of Nessler’s reagent was added and incubated at room temperature for 15 min. Then absorbance was measured at 430 nm in a multi-well plate reader (Biotek). For respective stress conditions, media (without inoculation) was taken as control. All the experiments were performed at least three times.

### Extracellular pH Measurement

The pH was measured as described in earlier literature (Gouzy et al., 2014). The samples, after growing under stress conditions, were harvested at 3,500 rpm. The resultant supernatant was collected in 50 ml tubes. For respective stress conditions, non-inoculated media was taken as control. All the experiments were performed at least three times.

### RNA Extraction and Semi-Quantitative RT-PCR

RNA extraction was performed by using the Trizol method as describe (Rizvi et al., 2019). After growing the cells under respective stress conditions, the samples were harvested and resuspended in trizol along with 0.1 mm glass beads. After lysing with bead beating, glycogen was added at room temperature (RT). Next, the mixture was vortexed vigorously after adding chloroform and then incubated at RT for 10 min. The upper aqueous layer was collected, and RNA was precipitated using isopropanol along with of glycogen. The obtained pellets were washed with 75% ethanol(v/v), and subsequently, air-dried at RT and dissolved in RNase-free water (Qiagen, Hilden, Germany). Any residual DNA contamination was removed by DNase treatment before reverse transcribing the RNA. The DNase treated RNA was further reverse transcribed with random hexamers as a primer using Superscript III Reverse Transcriptase (Invitrogen) to synthesize cDNA. With 1:10 diluted cDNA semi-quantitative RT-PCR was carried out for 24 cycles. Details of primers used are provided in Supplementary Table S2. Initial denaturation at 95°C (3 min), cycles of 95°C 15 s/appropriate temperature mentioned in the Supplementary Table S2; 20 s/72°C 20 s, final extension at 72°C for 10 min. 16S rRNA gene was used as an endogenous control. The products were fractionated on a 2% agarose gel and densitometric analysis was performed.

### Creation of the Genome-Scale Metabolic Model of *M.tb*

It was observed that none of the existing genome-scale metabolic models of *M.tb* encompassed the full set of 87 metabolites that were measured in our study (Supplementary Table S3). To address this problem, the recent and most comprehensive model of *M.tb* metabolism (viz. iEK1011) (Kavvas et al., 2018) was augmented with additional reactions to represent the metabolic states of *M.tb* best as observed in our experimental conditions. Evidence from literature and bioinformatic resources were gathered so that reactions corresponding to the missing metabolites could be added to the base model and any gaps in the model could also be filled with appropriate reactions. As a part of this process, two reactions in the pyrimidine metabolism, namely, CMP Nucleosidase (CMPN) and Cytosine Deaminase (CSND) were added. While there was genomic evidence for the former (enzyme encoded by Rv1205 and Rv2491), the latter was added during the gap filling step based on its presence in other mycobacterial species (e.g., *Mycobacterium* sp. JS623 and *Mycobacterium* sp. NRRL B-3805).

Additionally, protein sequences of *M.tb* were obtained from the NCBI database and subjected to analysis using PRIAM^2^ (Claudel-Renard et al., 2003). PRIAM allows automated enzyme detection by mapping the query sequence against PRIAM profiles. By the earlier literature (Chan et al., 2010), enzymes (EC numbers) with *e*-value < 10^–10^ were considered and were mapped against the BIOCYC database (Caspi et al., 2008) to obtain the corresponding reactions. Two reactions, namely, AP4A1 (Ap4A phosphorylase, EC 2.7.7.53, KEGG) and ADATT (ADP: ATP adenylyltransferase) (Mori et al., 2010) from purine metabolism, were thus added to update the iEK1011 model. The efficacy of the updated model was validated through an *in silico* single gene deletion analysis (see Supplementary Results).

### Flux Balance Analysis (FBA)

Flux balance analysis (FBA) is a widely used constraint-based approach for analyzing GEMs. It predicts metabolic fluxes by optimizing a given objective function (Orth et al., 2010). The commonly used objective functions include maximization of biomass (Fong et al., 2003; Schuster et al., 2008) minimization of total internal fluxes (Poolman et al., 2009; Poolman et al., 2013). Although significant advancements have been made in the development of computational approaches for an *in silico* metabolic analysis of both single cells as well as communities, there is not yet any tool which can reliably be used for utilizing static metabolite data into constrainment of metabolic models at a genomic scale. It is worthy to note in this regard that earlier attempts have been made translate metabolomic data into *in silico* fluxomic information (Cortassa et al., 2015). However, owing to the requirement of kinetic parameters, such attempts could only be applied to smaller subsystems and not ideal for genome scale models.

Subsequently, we explored the possibility of using modified versions of known FBA approaches to study metabolic changes in *M.tb*. While one of the methods relied on minimizing the total internal flux through the metabolic model, the other approach was based on optimizing flux through the objective function while constraining flux through a newly defined sink function (see Supplementary Methods and Supplementary Data Sheet S1). The inputs conditions for the flux analysis were appropriately designed so that they could mimic the actual experimental conditions. The conditions for Sauton’s media were replicated by allowing uptake of L-aspargine and ferric ammonium citrate [upto 1 mM/h/gdry weight]. Besides uptake of a small amount of glycerol and traces of other amino acids was also allowed. The latter was done with the objective of mimicking the initial state of the cells (before suspension in Sauton’s media). In the case of simulating the iron deficient condition, the uptake of ferric ammonium citrate was not allowed. Similarly, uptake of small amounts of H_2_O_2_ [upto 1 mM/h/g dry weight] was enforced on the *M.tb* model for simulating the oxidative stress condition. The input conditions while simulating nutrient starvation were the same as the control condition.

### Biosafety Committee Approval

All experiments were performed in the facilities (F-60) approved for *Mycobacterium* cultures by University of Hyderabad Institutional Biosafety Committee under the Department of Biotechnology, Govt. of India. The protocols of handling *Mycobacterium tuberculosis* for study in the laboratory were approved by the Institutional Biosafety Committee No. UH/SLS/IBSC/Review/SB-R-11 and SB-R-14.

## Results

### Alteration in Metabolic Profile and Metabolic Pathways of *M.tb* on Exposure to Stresses

**FIGURE 1:**
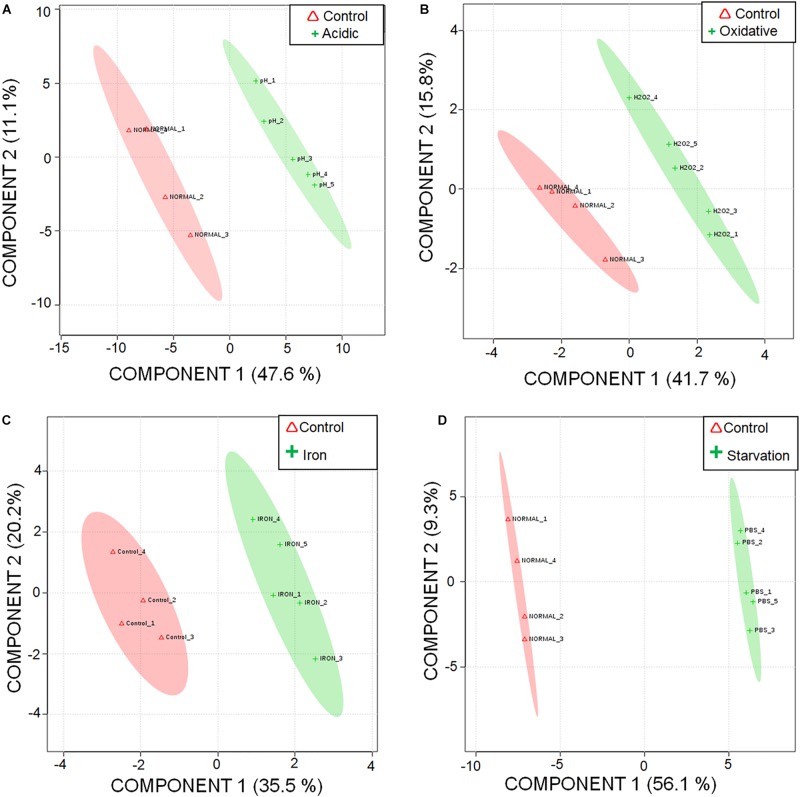
Segregation of metabolic profiles of acidic stress, oxidative stress, iron deprivation, and nutrient starvation from control using Partial Least Squares – Discriminant Analysis (PLS-DA). 2D score plot of **(A)** acidic stress vs. control; **(B)** oxidative stress vs. control; **(C)** iron deprivation (iron) vs. control; and **(D)** nutrient starvation vs. control are shown.

A total of 87 metabolites were detected consistently under different stresses (Supplementary Table S3) with a minimum base peak intensity of 30% in the LC-MRM/MS analyses (details in section Materials and Methods). The obtained results were subjected to statistical analyses using MetaboAnalyst (Xia et al., 2012). As compared to the control experiment, a total of 23, 6, 7 and 43 metabolites were quantified to be present at differential levels under acidic, oxidative, iron deprivation and nutrient starvation stresses respectively using a fold change cut-off of 1.5; *p*-value < 0.05; false discovery rate < 0.05 (Supplementary Table S4). A graphical representation of individual metabolite levels in different stresses is represented as a heat map in Supplementary Figure S1. Additionally, Partial Least Squares Discriminant Analysis (PLS-DA) was performed to explore the separation between the control and the stresses. While maximum segregations were observed in nutrient starvation and acidic stress, iron deprivation showed the least segregation from the control (Figures 1A–D). A possible reason for the latter could be the presence of available intracellular iron for utilization by *M.tb* despite exogenous iron deprivation. Since the stress duration was short, it is possible that *M.tb* continued to utilize its intracellular iron resources, thus showing metabolic profile nearly similar to control conditions.

**FIGURE 2:**
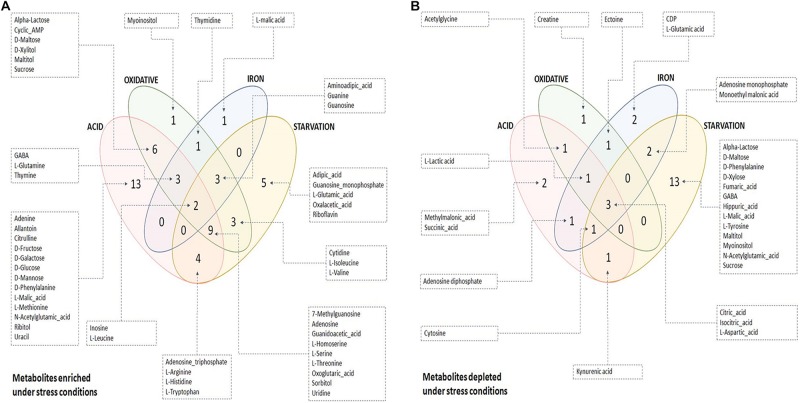
Euler diagrams depicting differentially abundant metabolites during stress conditions. **(A)** Metabolites enriched during stress with respect to control condition; **(B)** metabolites depleted during stress with respect to control condition. Only key metabolites differentiating between control and stress, as identified in PLS-DA analysis (VIP score ≥ 1), have been indicated in the diagram. The complete list of differentiating metabolites is provided in Supplementary Table S4.

The quality of the PLS-DA models as described in terms of goodness of fit (*R*^2^) and goodness of predictability (*Q*^2^) values is given in Supplementary Table S5. Metabolites differentiating control from each of the stresses were selected based on the Variable Importance in Projection (VIP) scores obtained from PLS-DA analysis. 47, 35, 21, and 46 metabolites with VIP score ≥ 1 were identified to be the major contributors for segregation of the model for acidic, oxidative, iron deprivation and nutrient starvation stresses respectively (Supplementary Figures S2A–D and Supplementary Table S4). The same is represented in the form of Euler diagrams in Figure 2. Significances of some of the observed metabolic changes during different stresses are discussed in subsequent sections.

**FIGURE 3:**
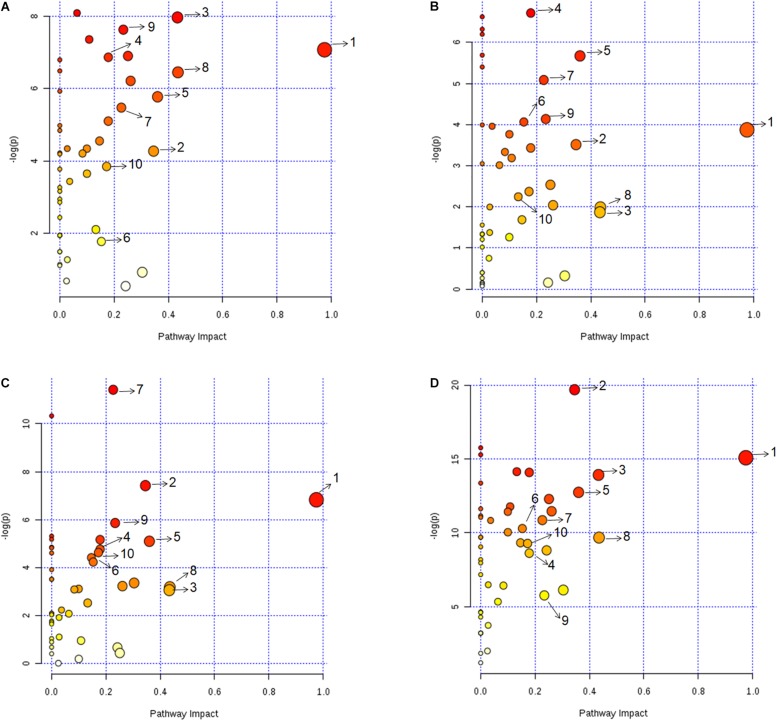
Metabolic pathway impact analysis revealing the significantly impacted metabolic pathways under **(A)** acidic, **(B)** oxidative, **(C)** iron deprivation, and **(D)** nutrient starvation stresses compared to the control. Each dot represents a unique metabolic pathway, with dot color corresponding to the –log(P) value and size of the dot corresponding to the pathway impact score. The same metabolic pathways across the four pairwise comparisons are represented by same letters. 1: Alanine, aspartate and glutamate metabolism, 2: Arginine and proline metabolism, 3: Citrate cycle (TCA cycle), 4: Cysteine and methionine metabolism, 5: Glycine, serine and threonine metabolism, 6: Nicotinate and nicotinamide metabolism, 7: Purine metabolism, 8: Pyrimidine metabolism, 9: Pyruvate metabolism, 10: D-Glutamine and D-glutamate metabolism. Detailed pairwise pathway impact is provided in Supplementary Table S6.

Metabolic pathway analysis was performed for pairwise comparison using MetaboAnalyst to obtain the biological significance of the changes in metabolite levels (Xia et al., 2012) (see section Materials and Methods). This is represented in Figure 3 where each dot represents a unique metabolic pathway, with the color of the dots corresponding to the -log(P) value and size of the dots corresponding to the pathway impact score. The same metabolic pathways across the four pairwise comparisons are represented by the same numbers (Figure 3). Six pathways that were found perturbed in acidic, iron deprivation and nutrient starvation stresses, were not affected significantly during oxidative stress. These were alanine, aspartate and glutamate metabolism, pyruvate metabolism, glutamine and glutamate metabolism, aminoacyl-tRNA biosynthesis, butanoate metabolism and arginine and proline metabolism. These pathways are collectively part of nitrogen assimilation and utilization (Gouzy et al., 2013; Majumdar et al., 2016). It is known that nitrogen metabolism pathway helps in alleviating acidic stress by producing ammonia (Gouzy et al., 2014). We make a similar observation in one of our earlier studies involving *M. smegmatis*, where several of these pathways were perturbed during acidic stress (Rizvi et al., 2019). Similarly, nutrient starvation can alter nitrogen assimilation and use efficiency to adjust to nutrient limiting conditions for survival. Our analysis also showed five pathways (inositol phosphate metabolism, fructose and mannose metabolism, riboflavin metabolism, glycolysis or gluconeogenesis) that were exclusively affected during nutrient starvation indicating re-wiring of central carbon metabolism to deal with nutrient deprivation and maintain energy homeostasis. Overall most affected pathways in our study belonged to nucleotide metabolism, amino acid metabolism, and central carbon metabolism (Supplementary Table S6). The assignment of different significance scores under different stress conditions to the same pathway suggested a distinct degree of perturbation in a pathway as a function of specific stress. Detailed pairwise pathway impact is provided in Supplementary Table S6.

### Flux Balance Analysis of *M.tb* Metabolism Under Different Stresses

Although pathway analysis of LC-MRM/MS data designated alterations in only a few pathways, it is expected that the manifestation of the effects of these perturbations on *M.tb* metabolism would spread across a wider array of metabolic pathways. To gain further insights, we adopted *in silico* methods to gauge the probable effects of these stresses on *M.tb* metabolism at a genome scale. The metabolic model of *M.tb* used in this study consisted of 1232 reactions involving 1014 enzyme-coding genes and 1000 metabolites (Supplementary Data Set S1). We designed two methods (henceforth referred to as FBA-M1 and FBA-M2) to constrain flux through *M.tb* GEM by modifying traditional flux balance analysis (FBA) approaches (see Supplementary Methods). Results obtained through the *in silico* simulations are presented in Supplementary Tables S7, S8 and in Supplementary Data Sheet S2. Data presented in Supplementary Data Sheet S2 may be visually analyzed through the Escher platform (Zachary et al., 2015).

While there were subtle quantitative variations in the results obtained through the two FBA approaches, insights obtained from both methods remarkably concur qualitatively at pathway/subsystem levels. Further, the results obtained from our *in silico* studies were in agreement with experimental findings published in earlier literature. For example, experimental evidence from published literature (Gouzy et al., 2014) suggest perturbations in amino acid metabolism, particularly the conversion of asparagine (Asn) to aspartate (Asp) during alleviation of acidic stress in *M.tb*. Similar trends were also noted in our *in silico* studies.

**FIGURE 4:**
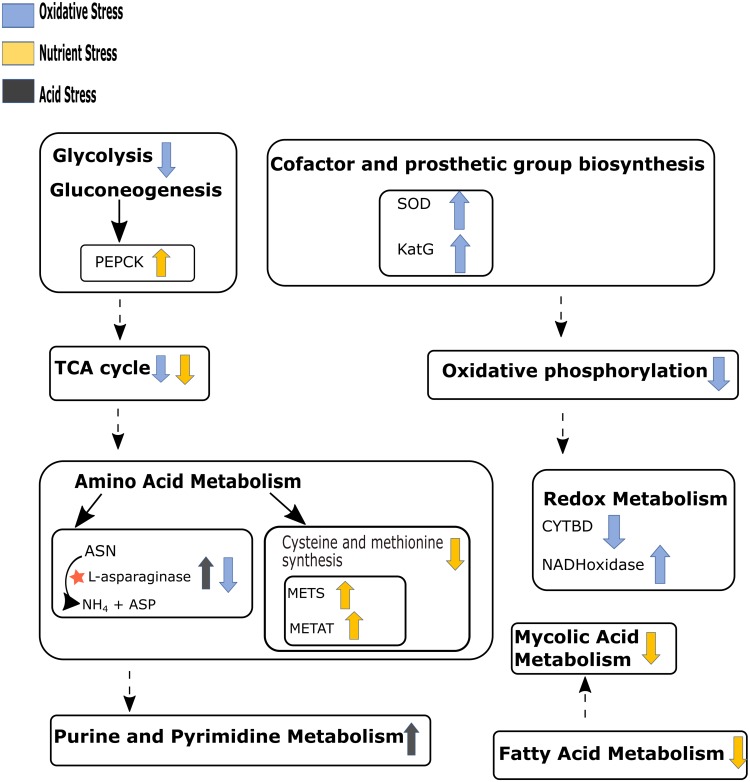
Schematic representation of the main pathways perturbed under different stresses as observed in the flux balance analysis (FBA) results. Up and down arrows beside the pathways’ name represent up-regulation and down-regulation respectively for that pathway. Arrow color code represents different stress conditions. Dashed arrows indicate multiple pathways between given two pathways. “★” shows maximum release of ammonia through asparaginase pathway under acid stress. PEPCK, phosphoenolpyruvate kinase; SOD, superoxide dismutase; KatG, catalase; METS, methionine synthase; METAT, methionine adenosyltransferase; CYTBD, cytochrome oxidase; ASN, aspargine; ASP, aspartate. Refer to Supplementary Figures S3–S6 for detail of reactions involved in the perturbed pathways. We have not included iron stress in this figure since there were no substantial changes observed.

Our *in silico* simulations showed down-regulation of mycolic acid synthesis pathway during nutrient starvation, which is in agreement with the existing literature (Jamet et al., 2015). Figure 4 summarizes the major pathway perturbations under varying stress conditions as observed in FBA results some of which are detailed as Supplementary Figures S3–S6 and discussed in the consequent paragraphs accordingly. The central carbon metabolism, nucleotide metabolism, and amino acid metabolism that were observed to be affected in our experimental conditions could also be replicated in the *in silico* analyses. While simulations also indicated perturbations in redox metabolism and oxidative phosphorylation during both acidic and oxidative stresses, folate metabolism was additionally altered during oxidative stress. Cofactor biosynthesis pathway was observed to be affected only during iron deprivation. The detoxification of H_2_O_2_ by catalase (KatG) was noted to be an essential reaction for the survival of *M.tb* during oxidative stress (Figure 4 and Supplementary Figure S3).

Further, an increased level of ammonia release was predicted during acidic stress (Supplementary Figures S5, S6). As compared to the other three stresses, a much higher number of reactions were predicted to be modified during nutrient starvations. As one would expect, all major metabolic pathways in *M.tb* seemed to be drastically slowed down under nutrient starvation stress. It included glycolysis/gluconeogenesis, TCA cycle, pentose phosphate pathway, glycerophospholipid metabolism, fatty acid metabolism, mycolic acid biosynthesis, amino acid metabolism, folate metabolism, riboflavin metabolism, oxidative phosphorylation and most reactions in nucleotide metabolism. Interesting, higher flux through certain reactions of the nucleotide salvage pathway (like NTD7 and NTD9) during nutrient starvation were noted. Both these reactions are intermediary processes leading to inosine (Ins) production. Pertinently, Ins modifications in DNA and RNA has previously been shown to be a highly regulated strategy among pathogens during stress conditions (Alseth et al., 2014). Another reaction which showed differential flux during nutrient starvation was mediated by phosphoenolpyruvate carboxykinase (PEPCK) wherein an increased flux favoring oxaloacetate (OAA) production from phosphoenolpyruvate (PEP), and CO_2_ was noted (Supplementary Figure S4). The role of this anaplerotic reaction during *in vivo* survival of *M.tb* has already been discussed in earlier literature (Beste et al., 2013).

### Transcripts Levels of Some of the Enzymes Involved in Metabolic Rewiring

We next checked if the differential levels of metabolites in pathways are a reflection of changes at transcript levels of enzymes in these pathways. We performed semi-quantitative RT-PCRs to monitor the expression levels of a few enzyme-encoding genes whose metabolic products were observed to be differential during various stress conditions. More precisely, the transcript levels of *GS, GDH, GOGAT, ureC, arcA, treS, glgB*, and *metK* genes were investigated during respective stresses (Supplementary Figure S7). Other than alterations in the levels of downstream metabolite products, we also observed some differences at the transcript levels of these genes during the stress conditions. Further, we observed that though urease activity differed during different stresses (Figure 6C), the transcript levels of ureC did not vary significantly during iron deprivation and acidic stress as compared to the control (Supplementary Figure S7). To further corroborate whether transcriptional changes play role in metabolic rewiring, we analyzed data from a previously published literature which had reported changes in *M.tb* gene expression under oxidative stress (Rodriguez et al., 2002). We observed that 32 genes which encoded for metabolic enzymes (and were present in our metabolic model) showed significant expression changes (adjusted *p*-value of 0.05). Further, only nine of them could be associated with the perturbed reactions in our *in silico* study (Supplementary Table S9).

## Discussion

The results obtained through experiments coupled with the *in silico* simulations pointed to a complex rewiring in *M.tb* metabolic network during stresses, with nutrient deprivation causing maximum perturbations, suggesting a noteworthy association of all cellular metabolites to the nutrient availability. Combining the two approaches, we also found increased flux through L-asparaginase that might be responsible for the generation of excess ammonia during acidic stress rather than increased activity of urease enzyme. Additionally, it could be suggested that proton-quenching through GABA shunt pathway or sugar alcohols can be possible mechanism to circumvent acidic and oxidative stresses. Utilizing excess of protons as a putative mechanism of stress response by *M.tb*, in the context of clues from existing literature, is discussed below. In the following sections, we also discuss the inferences drawn from FBA simulations using new approaches employed in this study that led to testable hypothesis such as, presence of a probable anaplerotic path for the generation of TCA intermediates from phosphoenolpyruvate using phosphoenolpyruvate kinase under nutrient stress and events of alternate use of cofactors by enzymes during oxidative stress response, showcasing *M.tb*’s potential to utilize multiple cofactors as stress-adaptation mechanism.

### Physiological Changes in *M.tb* During Adaptation to Stresses as Inferred From Metabolomics and Flux Balance Analyses

#### Energy Homeostasis

**FIGURE 5:**
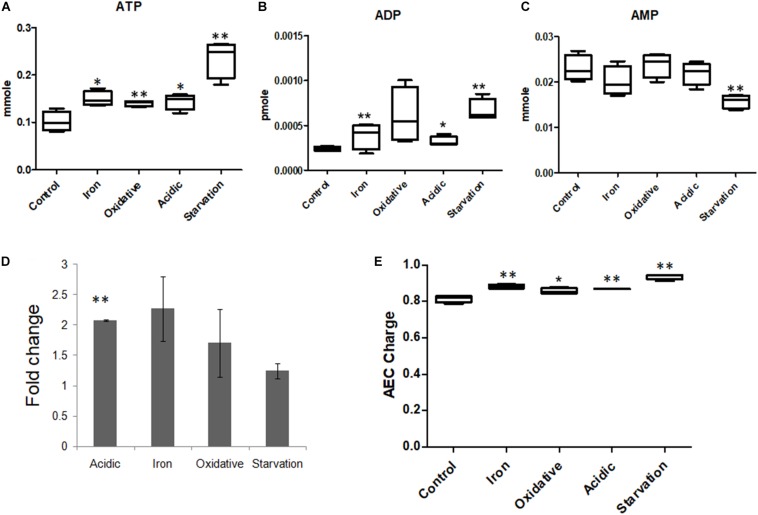
Adenosine nucleotide concentrations in *M.tb* during stresses. Measurements of the levels of **(A)** ATP, **(B)** ADP, and **(C)** AMP in *M.tb* on exposure to different microbicidal stresses. **(D)** The ratios of ATP/ADP represented as fold change compared to the control, and **(E)** adenylate energy charge (AEC) of *M.tb* under all stress conditions are also shown. ^∗∗^ Indicated *p*-value < 0.005 and ^∗^ indicated *p*-value < 0.05 as compared to control.

TCA cycle, which is known to be instrumental in energy generation as well as in providing biosynthetic precursors (Tian et al., 2005a; Mailloux et al., 2007) was affected in all stress conditions (Supplementary Figure S8). Metabolomics data showed that the levels of ATP, ADP and AMP were different in all stresses (Supplementary Table S4 and Figure 5), while the ATP/ADP ratio was highest for acidic and iron stress. Adenylate energy charge (AEC) values for control, acidic, oxidative, iron deprivation and nutrient starvation stresses were 0.81 ± 0.02, 0.87 ± 0.002, 0.86 ± 0.02, 0.88 ± 0.01, and 0.94 ± 0.01 (Figure 5). It was therefore clear that though large fluctuations in the ATP, ADP, and AMP concentrations were observed during different stresses, the AEC values were maintained close to that of control indicating adjustments in energy dynamics for stress adaptation in *M.tb*. ATP that was observed to accumulate during nutrient starvation in our experiments could be explained by FBA results that showed a decrease in the flux through the major energy consuming metabolic pathways, such as mycolic acid synthesis, membrane metabolism and fatty acid metabolism (Supplementary Figure S4). Thus, the original pool of ATP and ADP which was present within the cell before being transferred from nutrient sufficient media (control) to nutrient deficient media (nutrient starvation) largely remained underutilized due to lack of substrates to act. These results for the first time highlighted the ability of *M.tb* to adjust its energy dynamics in terms of maintaining AEC within fine physiological ranges to sustain functional metabolic viability for immediate adaptation to environmental stresses.

#### Accumulated α-Keto-Glutarate (AKG) During Nutrient Starvation and Acidic Stress May Have Different Fates for Adequate Adaptive Responses

Amongst TCA cycle metabolites, a notable increase in α-keto-glutarate (AKG) (also referred to as oxoglutaric acid) levels were observed in all stresses, especially during nutrient starvation. One possible source of AKG is glutamate, catalyzed by enzyme glutamate dehydrogenase (GDH) (Supplementary Figure S5). This anaplerosis node may serve as a universally important step for early metabolic adaptation during stresses and has previously been suggested to have importance in overcoming acidic, oxidative, and nitrosative stresses (Gallant et al., 2016). GDH in the process of converting glutamate to AKG releases ammonia and also reduces NAD+ to NADH (Maksymiuk et al., 2015). Assuming that the predicted accumulation of AKG should translate into the release of ammonia, we attempted to score ammonia formation during different stresses through FBA simulations. Our FBA simulations predicted that ammonia was negligibly formed during nutrient starvation, not formed during oxidative stress but was highly produced during acid stress. The reaction of glutamate converting to AKG by GDH was found to be marginally down-regulated during nutrient starvation (Supplementary Tables S7, S8). A possible explanation for this down-regulation could be the limited availability of Asp. While the growth media was Asp limiting, flux through L-asparaginase (ASNN) mediated conversion of Asn to Asp during nutrient starvation was also found to be low. Instead, the accumulation of AKG during nutrient starvation could probably be attributed to altered flux through the reaction catalyzed by aspartate transaminase (ASPTA), wherein turnover of AKG to oxaloacetic acid (OAA) was predicted to be lower than normal. The lower turnover of OAA was however compensated by anaplerosis by PEPCK. As mentioned earlier, during nutrient starvation, the conversion of PEP to OAA was observed to be active by simulations (Supplementary Figure S4). The same is important during *in vivo* survival of *M.tb* in an earlier study (Beste et al., 2013). On the contrary, during oxidative stress, GDH mediated reaction was found to operate in reverse direction wherein ammonia was consumed and also showed drastic down-regulation in the flux. ASNN mediated reaction also showed around 90% decrease in the flux compared to control (Supplementary Table S7).

Interestingly, flux leading to AKG was also observed in certain other reactions during acidic stress. However, the mechanism of AKG accumulation and utilization was predicted to be different during acidic stress as compared to nutrient starvation. The measured concentration of Asp and Glu were low, which was probably suggestive of higher utilization of these amino acids. Concomitantly, flux through reactions leading to the generation of AKG and ammonia from Asn was higher (Supplementary Figure S6).

**FIGURE 6:**
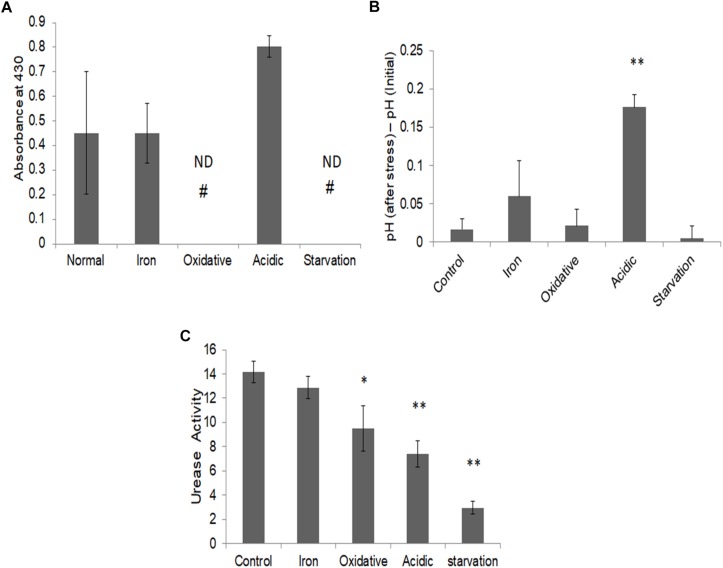
Ammonia release and alleviation of stress. **(A)** Ammonia estimation post stress in the respective culture media supernatant using Nessler’s assay as assessed by absorbance at 430 nm normalized to respective blank; **(B)** Measurement of change in pH in *M.tb* culture post stresses; and **(C)** urease activity (in terms of ammonia nmol/mg protein/min) in *M.tb* cell lysates post stress measured by using urease activity assay kit (ab204697, Abcam Plc., United Kingdom). ^∗∗^Indicated *p*-value < 0.005 and ^∗^ indicated *p*-value < 0.05 as compared to control. # ND, not detectable.

To validate this prediction, we measured the released ammonia in culture media during different stresses (Figure 6A). While a basal level of ammonia was found to be released during iron deprivation, it was nearly twofold elevated during acidic stress. Ammonia levels could not be detected under oxidative and nutrient starvation conditions. Thus, as predicted by FBA simulations, elevated levels of ammonia were indeed released by *M.tb* during acidic stress and negligible during nutrient starvation or oxidative stress. To test, if the released ammonia is also associated with alleviation of acidic stress, the pH levels of the cell cultures, pre- and post-stress were measured (Figure 6B). The pH of the media was seen to rise significantly only during acidic stress (Figure 6B). These experiments supported our *in silico* predictions about the release of ammonia being significant only in response to acidic stress.

Based on these observations, pathways with the potential to release ammonia were further probed for acidic stress. Conventionally, the urea cycle is believed to be predominantly responsible for adaptation to acidic stress in bacteria (Mobley et al., 1995; Mendz and Hazell, 1996). To check if urease is responsible for the ammonia production in these conditions, the enzymatic activity of urease was measured during different stresses. To our surprise, we observed a decrease in urease activity in all stresses as compared to the control (Figure 6C). This suggested that urea cycle under acidic stress may not be principally responsible for increased ammonia in the culture supernatant in our experimental conditions. We once again reverted to our FBA results, which suggested increased flux through L-asparaginase (ASNN) reaction, which may be responsible for the generation of excess ammonia during acidic stress (Supplementary Figure S6). Generation of ammonia during the conversion of asparagine to aspartate through reactions catalyzed by L-asparaginase, as a strategy to mitigate low pH in *M.tb* has also been advocated in recent literature (Gouzy et al., 2013). Collectively, with this, we could demonstrate differential adjustments of metabolic networks concerning α-keto-glutarate (AKG) utilization in different stresses in *M.tb*.

#### Metabolomics Data and FBA Simulations Pointed to Proton Quenching as a Possible Mechanism to Counter Acid Stress

A set of the metabolites identified to be differentially abundant during various stresses were that of GABA shunt pathway as presented schematically along with their differential levels in Supplementary Figure S9. When glutamate gets metabolized to GABA by glutamate decarboxylase, a proton is utilized, which under acidic stress may prove beneficial for a cell (Supplementary Figure S9). Apart from removing proton at low pH, GABA shunt can also reduce NAD+ to form succinate by metabolizing succinate semialdehyde by the action of succinate-semialdehyde dehydrogenase (Tian et al., 2005a). Thus, from this study, we may infer that GABA can quench protons and at the same time participate in NAD+/NADH balance, thereby contributing to the early adaptations of *M.tb* during both acidic and oxidative stresses. The study also encourages further investigation of this less explored pathway in understanding *M.tb* pathogenesis.

Further, we also observed high levels of sugar alcohol during stresses, which are usually generated through the pentose phosphate pathway (PPP) (Lin, 1988; Toivari et al., 2007). PPP is one of the fundamental pathways known for providing reducing molecules for metabolism, overcoming oxidative stress, maintaining carbon homeostasis, and supplying precursors for amino acid as well as nucleotide biosynthesis (Stincone et al., 2015). Sugar alcohols are reduced form of an aldose or ketose sugar and their conversion from sugar to sugar alcohol consumes excess protons from the milieu while utilizing NADH. In our data, we observed an exceptional increase in sugar alcohols, xylitol (25-fold) and ribitol (21-fold) in acidic stress and a modest increase in xylitol and ribitol concentrations in oxidative and iron deprivation stresses (Supplementary Table S4). However, during nutrient starvation, the levels of xylitol and ribitol were lower as compared to control (0.76-fold and 0.79-fold, respectively). The differential levels of xylitol and ribitol in different stresses suggested a greater impact of PPP pathway in acidic stress as compared to oxidative and iron stresses. This hypothesis is further supported by higher flux through Ribulose-5-phosphate metabolizing reactions RPI and RPE during acid stress. The drastic accumulation of sugar alcohols including xylitol and ribitol in acid stress is suggestive of their possible involvement in the metabolic adaption of *M.tb* to counter acidic stress, which has not been studied in detail. Notably, xylitol is a known bacteriostatic metabolite. Although it is taken up by many bacteria, they are unable to metabolize xylitol. Xylitol is also known to inhibit bacterial metabolism, including acid production (Ohashi et al., 1991; Trahan et al., 1991; Roberts et al., 2002; Nayak et al., 2014). This possibly explains potential adaptation to acid stress, wherein the acid generating pathways are blocked or re-routed in the presence of elevated levels of xylitol. With these observations, we propose that yet another mechanism by which *M.tb* may resist acidic stress is by utilizing excess protons.

#### Multiple Regulations on Enzymes During Metabolic Adjustments During Stress Adaptation

Some of our earlier studies which inspected the cross-talks between metabolic network, gene regulatory network, and host-pathogen interaction network of *M.tb* during hypoxia (Bose et al., 2018) had suggested changes at the transcript levels of genes as a probable mechanism aiding in such metabolic rewiring. When we checked the expression levels of some selected enzyme-coding genes by semi-quantitative RT-PCRs, few, but not all, showed significant differences at the transcript levels as response to stresses (Supplementary Figure S7). Urease activity, but not the transcript levels of ureC (Supplementary Figure S7), varied significantly during acidic stress as compared to the control, in our conditions (Figure 6C). It was therefore clear that the regulation of the enzymes during initial stages of stress responses, besides changes at transcription levels, can also be controlled through regulation of enzymatic activities by a combination of intrinsic and extrinsic factors. Such changes may be mediated through various factors including changes in thermodynamic conditions, alternate use of cofactors, etc. We analyzed our results for events wherein alternate metabolic pathways were invoked due to differential use of cofactors (Supplementary Table S10). Most events of alternate use of metabolites were observed for oxidative stress. One such example is the use of menaquinol over ubiquinone as a cofactor to NADH dehydrogenase under oxidative stress. Effects of the use of such alternate pathways, especially during stress alleviation in bacterial systems have been elucidated in earlier literature (Shimizu, 2013; Armingol et al., 2018). However, very few such events were noted for the stresses other than oxidative stress for *M.tb*. Therefore, it is likely, that although cofactors play a part in metabolic rewiring, there are other factors, such as post-translational modifications (Oliveira and Sauer, 2012), which may also contribute significantly to enzyme activity and assembly and thereby changes in metabolic rewiring.

### Limitations of the Present Study

The study was limited to identification of 108 metabolites available as standards due to which several metabolites including lipids were not identified. Despite a limited number of metabolites that could be measured by LC-MS/MRM from the entire pool of metabolites owing to these technical limitations, mathematical tools like FBA was used to overcome such constraints. It may, however, be noted that FBA comes with certain limitations (Orth et al., 2010). Firstly, it does not use kinetic parameters and is mostly applicable for determining fluxes at steady state. Secondly, it does not account for regulatory effects such as activation of enzymes or regulation of gene expression. So, its predictions may not always be accurate specifically in situations where short term regulations take place. However, time-dependence and environmental transitions may be (partially) accounted in FBA analysis through the use of appropriate constraints derived from experimental or literature evidences. Such constrainments may be in the form of gene expression data, protein or metabolite abundances, etc. (Cortassa et al., 2015; Bose et al., 2018).

## Conclusion

In this paper, we have discussed approaches to comprehend the genome-scale metabolic changes in *M.tb* during early stress adaptation from a small subset of measured metabolite concentrations. Using *in silico* approaches, we were able to predict the probable alterations in the paths of the flow of metabolic fluxes, thereby leading to adaptation to stresses. Understanding that *in silico* method has limitations due to the incompleteness of the genome-scale metabolic models and non-availability of thermodynamic parameters for all the enzymatic reactions in case of *M.tb*, efforts were made to improve the existing *M.tb* metabolic model with regards to the metabolites which were measured through LC-MRM/MS. In spite of our best efforts, we were unable to capture all the measured metabolites in the *M.tb* model. In particular, metabolites from the sugar alcohol metabolic pathway could not be captured. Thus, there exists an opportunity to improve the *in silico* model of *M.tb* metabolism. While this study was a single time-point study, a time-course experiment would provide better perception on how the mycobacterial system responds to these stresses over time for early adaptation. Such studies and leads from these observations are under investigations. Conclusively, with this study, together with earlier reports (Gouzy et al., 2013, 2014), we propose glutamate dehydrogenase (GDH), glutamate synthetase (GS) and glutamine oxoglutarate aminotransferase (GOGAT) as important nodes for early adaptation to microbicidal stresses, especially during acidic stresses. In addition, GABA shunt and sugar alcohols synthesis pathways are attractive leads to understand the pathobiology of *M.tb*.

## Data Availability Statement

All datasets generated for this study are included in the manuscript/Supplementary Files.

## Author Contributions

AR, TM, KB, and LM performed the experiments. AC and AS performed the *in silico* analyses. AR, AS, AC, TB, AD, SSM, SB, and SCM analyzed the data and wrote the manuscript. SR, SSM, SB, and SCM supervised the project.

## Conflict of Interest

SSM, AD, TB, and AS were employed by Tata Consultancy Services Ltd. The remaining authors declare that the research was conducted in the absence of any commercial or financial relationships that could be construed as a potential conflict of interest.

## Footnotes

1 www.metaboanalyst.ca/MetaboAnalyst

2 http://priam.prabi.fr/
