# Supplementary material for: Rewiring of Metabolic Network in Mycobacterium tuberculosis During Adaptation to Different Stresses
Source: Front Microbiol. 2019 Oct 29;10:2417. doi: 10.3389/fmicb.2019.02417 (PMC6828651; doi:10.3389/fmicb.2019.02417)
Supplement: Supplementary file 3 [file Data_Sheet_1.PDF]

Data sheet 1

Computational approaches adopted for Flux Balance Analysis (FBA)

Method for FBA-M1

In general, FBA considers a system to be at a steady state in which the rate of production of each internal metabolite in the network is equal to its rate of consumption. This state can be represented mathematically as:

$$S.V = 0$$

where, **V** is a vector of fluxes through the metabolic network and **S** is the stoichiometry matrix (Lee et al., 2006). **S** is an M × N matrix where **M** represents all the metabolites (in rows), and **N** represents the reactions (in columns) in the network. To reproduce the experimental conditions through FBA, the flux through certain metabolic reactions in the *M.tb* model were constrained using the metabolite concentrations which were measured for *M.tb* under different stress conditions. For this purpose reactions about metabolites which showed significant fold changes during stress (**Table S4**) and which were present in the *M.tb* model were chosen. For example, the concentration of inosine (Ins) was found to be over 7-fold higher in acid stress condition as compared to the control condition. Accordingly, the lower bound (LB) of flux through adenosine deaminase (ADA) reaction (the primary source of Ins in the *M.tb* model) was constrained to 0.05mM/gDW/h while simulating acid stress. The constrainment was based on the following formulae:

$$LB(stress) = LB(control) \times \left(1 + \frac{\%increase \in conc.ofmetaboliteunderstress}{100}\right)$$

In cases where the metabolites were absent in control condition but were measured under stress conditions, the lower bound was set as 0.001mM/gDW/h to allow production of that particular metabolite under defined stress condition. The flux through the biomass function was constrained based on the experimentally observed culture OD values (**Table S11**). The list of constrained reactions for each of the stress conditions has been presented in Table S12.

It may be noted that while simulating for the oxidative stress condition, uptake of H_2O_2 was allowed in the model by adding a transporter for hydrogen peroxide (H_2O_2tex). Also, a superoxide-generating reaction (NADH oxidase) was added in the model to mimic oxidative stress *in silico*.

Gnu Linear Programming Kit (http://www.gnu.org/software/glpk/) and linear programming (LP) based ScrumPy metabolic modeling package (Poolman, 2006) for the simulation. The LP was defined as:

$$minimiseZ = \sum_{i=1}^{N} c_i . v_i = C^T.V$$

$$subjectS.V = 0$$

$$LB \leq v_i \leq UB$$

where, **Z** is the objective function, **V** is the vector of fluxes through reactions (v_i), C is the transpose of a vector of objective coefficients (c_i), **LB** and **UB** are the vectors of fluxes lower bounds and upper bounds, respectively. The objective function used in the analysis was a minimization of the total cellular flux. Changes in fluxes (with respect to the control condition) through the metabolic reactions in the *M.tb* model were measured for four different stress conditions – acidic, oxidative, iron stress and nutrient starvation.

Method for FBA-M2

Studies have shown that enforcing multiple constraints or secondary objectives (Schuetz et al., 2007; Maarleveld et al., 2015) improve the accuracy of flux balance analysis, particularly constraints from omics data. Existing methods for metabolomics data integration rely on time series measurements (Mahadevan et al., 2002; Bordbar et al., 2017), kinetic parameters, thermodynamic constraints (Hoppe et al., 2007; Haraldsdottir et al., 2012; Noor et al., 2013) or qualitative bounds (Schmidt et al., 2013). FBA-M2 was developed as a method to integrate experimentally measured static metabolite concentration data (from case-control studies) into genome-scale metabolic models (GEMs) for FBA. The measured intracellular metabolite concentration data is used to mimic a given physiological state (say, stress condition) by way of a secondary objective to observe the changes in the steady-state flux predictions. The secondary objective function is defined as:

$$v_{accum} = \sum_{i=1}^{n} c_i \, . \, m_i$$

where, **n** is the number of metabolites whose concentrations were measured, **m∈M** where **M** represents all metabolites in the network, $\mathbf{c_i}$ is the measured concentration of metabolite normalized to cell count in mM units, and $\mathbf{v_{acuum}}$ is the flux through sink function or the secondary objective. The LP was defined as:

$$maximise Z = v_{accum}$$

$$subject v_{LB,biomass} = v_{biomass} \times (f \vee gr)$$

$$subject S.V = 0$$

$$LB \leq v_i \leq UB$$

where, **a** $\mathbf{v_{acuum}}$ is the flux through sink function, $\mathbf{v_{LB,\ biomass}}$ is the flux through sink function is the lower bound for the biomass flux, **gr** is the experimentally measured growth rate, and **f** is the fraction of growth rate relaxation (based on experimental or theoretical data).

This above formulation is a multi-component objective (biomass and measured metabolite concentrations) constrainment that adjusts the flux through the metabolic model to best mimic the environmental conditions. Validation results showed that if exact growth conditions (in the form of nutrient uptake rates and/or growth rates) are mimicked, maximization of the multi-component objective could predict very accurate steady-state fluxes in an *Escherichia coli* metabolic model (**Table S13**). Given that exact nutrient uptake or growth rates were not known in the present study, a parsimonious (minimization) method was used to obtain nutrient uptake

rates and biomass fluxes within biologically feasible value, while optimizing the multi-component objective using measured metabolite concentrations and culture OD values (**Table S3 and Table S11** respectively). Further, the LB of flux through the secondary objective was assumed using a linear relationship of accumulation of the most abundant metabolite present in the central carbon metabolism, i.e. pyruvate. The concentration of pyruvate measured at the end of the experiment was divided by the total time of growth to arrive at a minimum rate for the secondary objective.

Supplementary Results

Validation of the iEK1011-mod model of *Mycobacterium tuberculosis* H37Rv (*M.tb*) metabolism

The iEK1011 (Kavvas et al., 2018) is the most comprehensive metabolic reconstruction model of *M.tb* that is available to date. However, it was observed that reactions associated with some of the metabolites that were measured in our study (**Table S3**) were not present in the iEK1011 model. Therefore, four additional reactions (see Appendix Supplementary Methods section) were added to the iEK1011 model (henceforth referred to as the iEK1011-mod model) to better represent the metabolic state of the *M.tb* cells under stress conditions. The final model consists of 1232 reactions and 1014 genes. An *in silico* single gene deletion analysis was performed to compare the correctness of the updated model with data from experimentally observed gene essentiality studies as reported in the original reconstruction (Kavvas et al., 2018). Results (**Table S14**) showed that the performance of the iEK1011-mod model did not significantly deviate from the original iEK1011 model. However, in spite of no significant loss in accuracy, the iEK1011-mod model (presented herein) encompassed a higher number of metabolites which were measured in this study. It may, therefore, be assumed that the iEK1011-mod model would be able to mimic the metabolic state of the *M.tb* cells with greater reliability.

Validation of FBA-M2

To test the accuracy of the FBA-M2 approach an existing *E.coli* omics dataset (Ishii, et al., 2007) that included both fluxomics and metabolic concentration measurements was used. Given the non-availability of a comparable method which uses experimentally measured metabolic data to constrain flux through (genome-scale) metabolic reconstructions, we benchmarked the performance of the FBA-M2 approach against classical FBA methods and those which uses transcriptomic data for flux constrainment (Schmidt et al., 2013; Machado and Herrgard, 2014; Song et al., 2014). The methods and code (other than Classical Flux Balance Analysis and FBA-M2) were obtained from previously published papers (Machado and Herrgard, 2014; Song et al., 2014). The *E.coli* model reconstruction iJO1366 (Song et al., 2014) was used for the validation purpose. Results of the analysis are presented in **Table S13**. The RMSD values obtained for each of the methods indicate that FBA-M2 could replicate the experimental observations with better accuracy as compared to any of the existing methods. Overall, the results obtained from this analysis established the acceptability of FBA-M2 as an *in silico* method for studying flux through metabolic reactions in genome-scale networks using measured metabolite data.

References

Bordbar, A., Yurkovich, J.T., Paglia, G., Rolfsson, O., Sigurjonsson, O.E., and Palsson, B.O. (2017). Elucidating dynamic metabolic physiology through network integration of quantitative time-course metabolomics. *Sci Rep* 7**,** 46249. doi: srep46249 [pii] 10.1038/srep46249.

Haraldsdottir, H.S., Thiele, I., and Fleming, R.M. (2012). Quantitative assignment of reaction directionality in a multicompartmental human metabolic reconstruction. *Biophys J* 102(8)**,** 1703-1711. doi: S0006-3495(12)00263-9 [pii] 10.1016/j.bpj.2012.02.032.

Hoppe, A., Hoffmann, S., and Holzhutter, H.G. (2007). Including metabolite concentrations into flux balance analysis: thermodynamic realizability as a constraint on flux distributions in metabolic networks. *BMC Syst Biol* 1**,** 23. doi: 1752-0509-1-23 [pii]10.1186/1752-0509-1-23.

Kavvas, E.S., Seif, Y., Yurkovich, J.T., Norsigian, C., Poudel, S., Greenwald, W.W., et al. (2018). Updated and standardized genome-scale reconstruction of *Mycobacterium tuberculosis* H37Rv, iEK1011, simulates flux states indicative of physiological conditions. *BMC Syst Biol* 12(1)**,** 25. doi: 10.1186/s12918-018-0557-y 10.1186/s12918-018-0557-y [pii].

Lee, J.M., Gianchandani, E.P., and Papin, J.A. (2006). Flux balance analysis in the era of metabolomics. *Brief Bioinform* 7(2)**,** 140-150. doi: 10.1093/bib/bbl007.

Maarleveld, T.R., Wortel, M.T., Olivier, B.G., Teusink, B., and Bruggeman, F.J. (2015). Interplay between constraints, objectives, and optimality for genome-scale stoichiometric models. *PLoS Comput Biol* 11(4)**,** e1004166. doi: 10.1371/journal.pcbi.1004166 PCOMPBIOL-D-14-01699 [pii].

Machado, D., and Herrgard, M. (2014). Systematic evaluation of methods for integration of transcriptomic data into constraint-based models of metabolism. *PLoS Comput Biol* 10(4)**,** e1003580. doi: 10.1371/journal.pcbi.1003580 PCOMPBIOL-D-13-02075 [pii].

Mahadevan, R., Edwards, J.S., and Doyle, F.J., 3rd (2002). Dynamic flux balance analysis of diauxic growth in Escherichia coli. *Biophys J* 83(3)**,** 1331-1340. doi: S0006-3495(02)73903-9 [pii]10.1016/S0006-3495(02)73903-9.

Noor, E., Haraldsdottir, H.S., Milo, R., and Fleming, R.M. (2013). Consistent estimation of Gibbs energy using component contributions. *PLoS Comput Biol* 9(7)**,** e1003098. doi: 10.1371/journal.pcbi.1003098 PCOMPBIOL-D-12-01961 [pii].

Poolman, M.G. (2006). ScrumPy: metabolic modelling with Python. *Syst Biol (Stevenage)* 153(5)**,** 375-378.

Schmidt, B.J., Ebrahim, A., Metz, T.O., Adkins, J.N., Palsson, B.O., and Hyduke, D.R. (2013). GIM3E: condition-specific models of cellular metabolism developed from

metabolomics and expression data. *Bioinformatics* 29(22)**,** 2900-2908. doi: btt493 [pii]10.1093/bioinformatics/btt493.

Schuetz, R., Kuepfer, L., and Sauer, U. (2007). Systematic evaluation of objective functions for predicting intracellular fluxes in Escherichia coli. *Mol Syst Biol* 3**,** 119. doi: msb4100162 [pii] 10.1038/msb4100162.

Song, H.S., Reifman, J., and Wallqvist, A. (2014). Prediction of metabolic flux distribution from gene expression data based on the flux minimization principle. *PLoS One* 9(11)**,** e112524. doi: 10.1371/journal.pone.0112524 PONE-D-14-35007 [pii].

Supplementary Figures

Figure S1

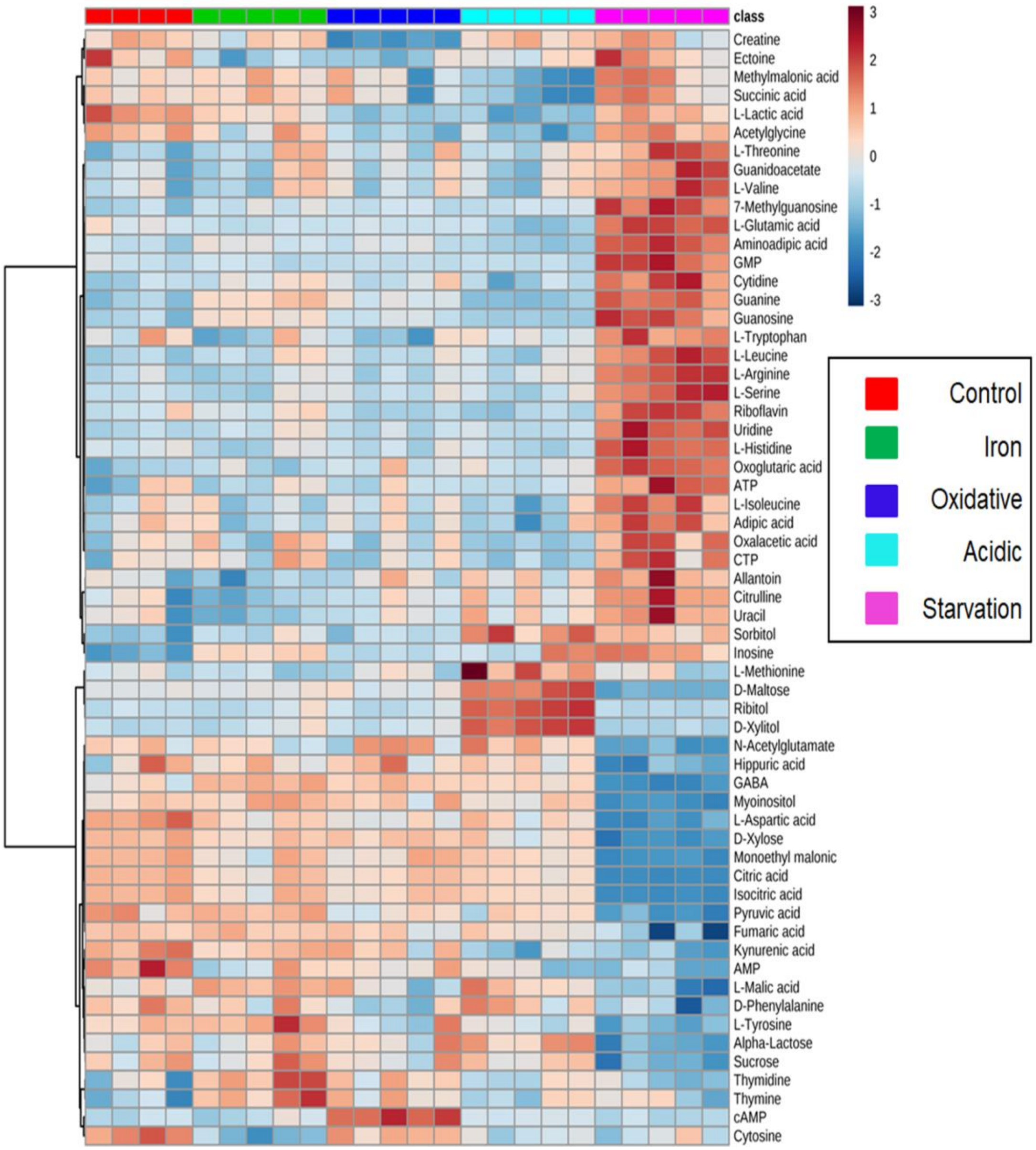

Figure S1: Heat map representation of the metabolic changes in *M.tb* induced by acidic, oxidative, iron deprivation (iron), and nutrition starvation stresses. Individual samples are represented on the horizontal axis, and metabolites are on the vertical axis. Each stress is represented by colors as indexed in the figure. Four replicates for control and five replicates for each stress were used for analyses. The color gradient in the heat map (blue to red) indicates the relative concentration of metabolites across different stresses and technical replicates. Normalization was performed using the following calculation [Normalized metabolite peak area for Stress Experiment = (Ps / ODs) X ODc], where P is the peak area, OD is absorbance at 600nm. Refer Table S3.

Figure S2

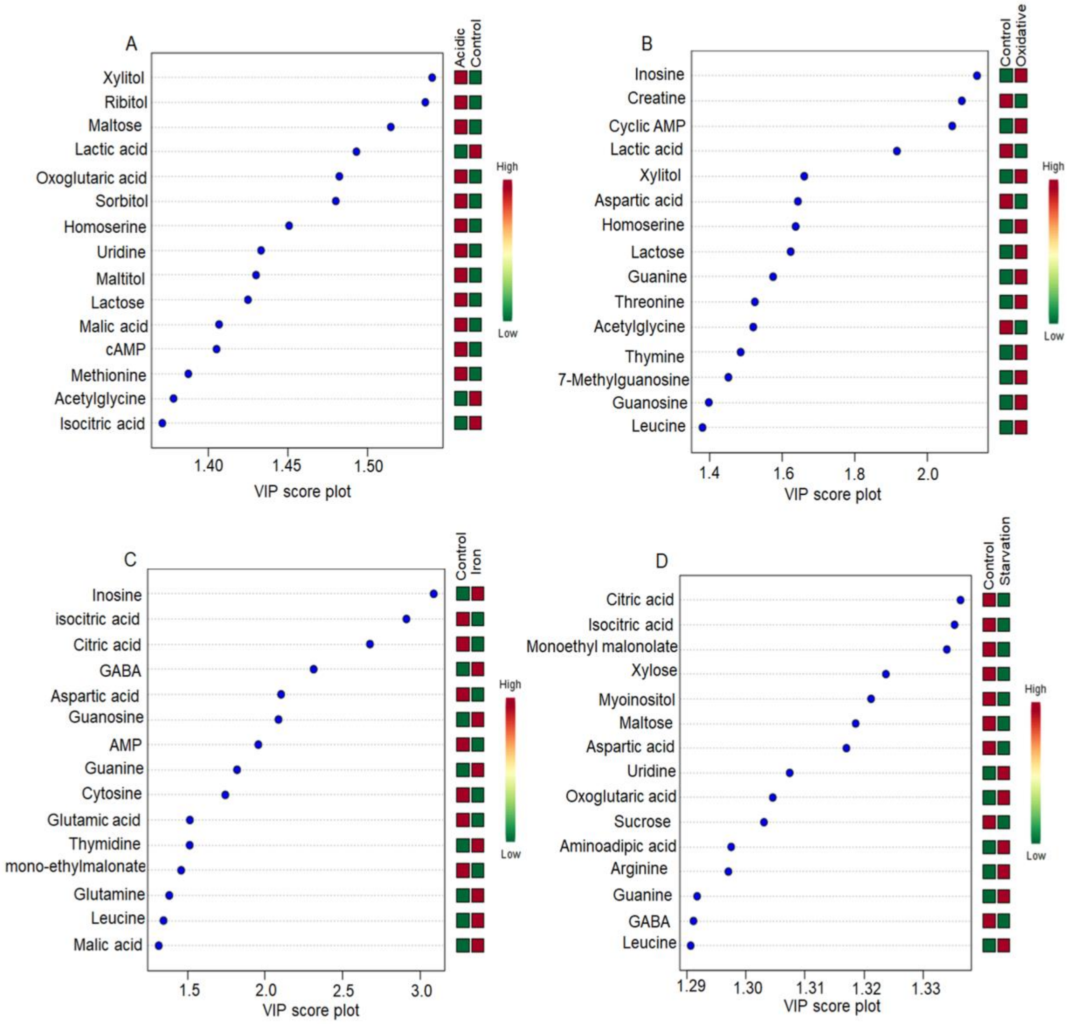

Figure S2: Variable Importance in Projection (VIP) plots. VIP plots indicating the most discriminating metabolite (A) for acidic stress *vs* control; (B) for oxidative stress *vs* control; (C) for iron deprivation (iron) *vs* control; (D) for nutrient starvation *vs* control. Also refer main figures 1 and 2 and Table S4.

Figure S3

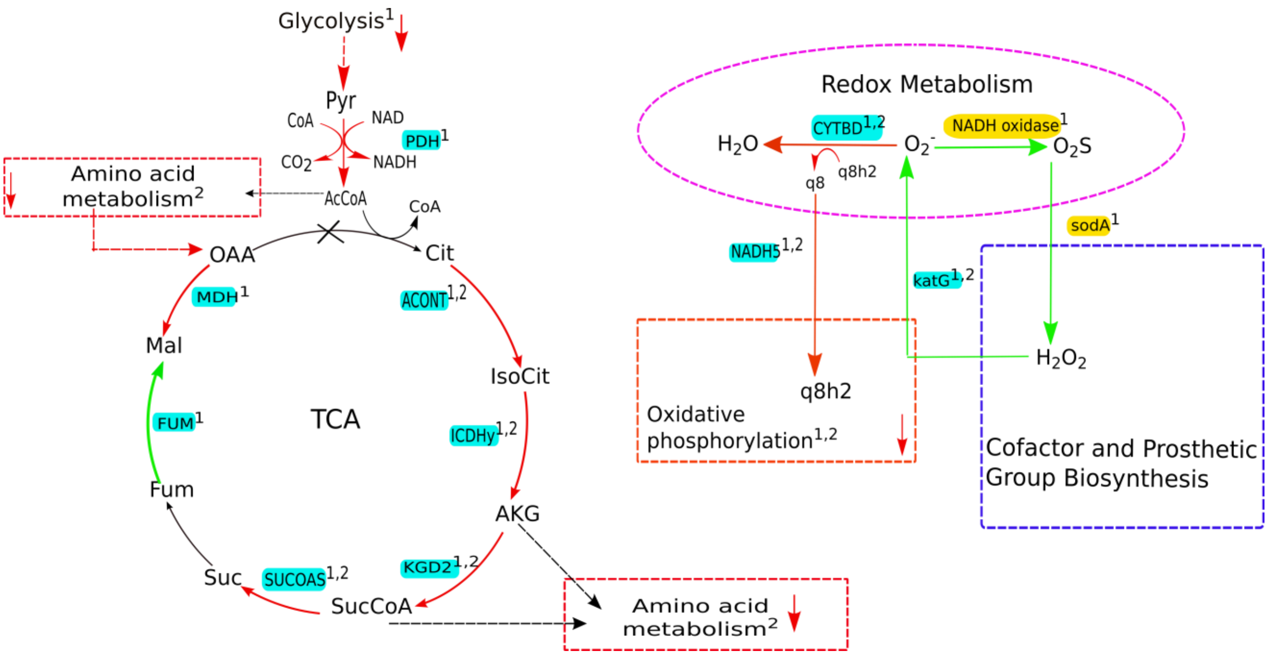

Figure S3. *In-silico* simulation of *M.tb* GEM for oxidative stress

Notable perturbations were observed in TCA, redox metabolism and oxidative phosphorylation. The detoxification of H_2O_2 by catalase (katG) was noted to be an essential reaction. Red arrows indicate a decrease in flux through the corresponding pathway/reaction when compared to the control flux. NADH oxidase and superoxide dismutase (sodA) were found to become active only under oxidative stress. Green arrows indicate an increase in flux through the corresponding pathway/reaction when compared to the control flux. Oxaloacetate (OAA) to citrate (Cit) conversion became inactive under oxidative stress. 1 and 2 in superscript indicate the findings in FBA-M1 and FBA-M2 respectively. Refer Figure 4 in the main text.

Figure S4

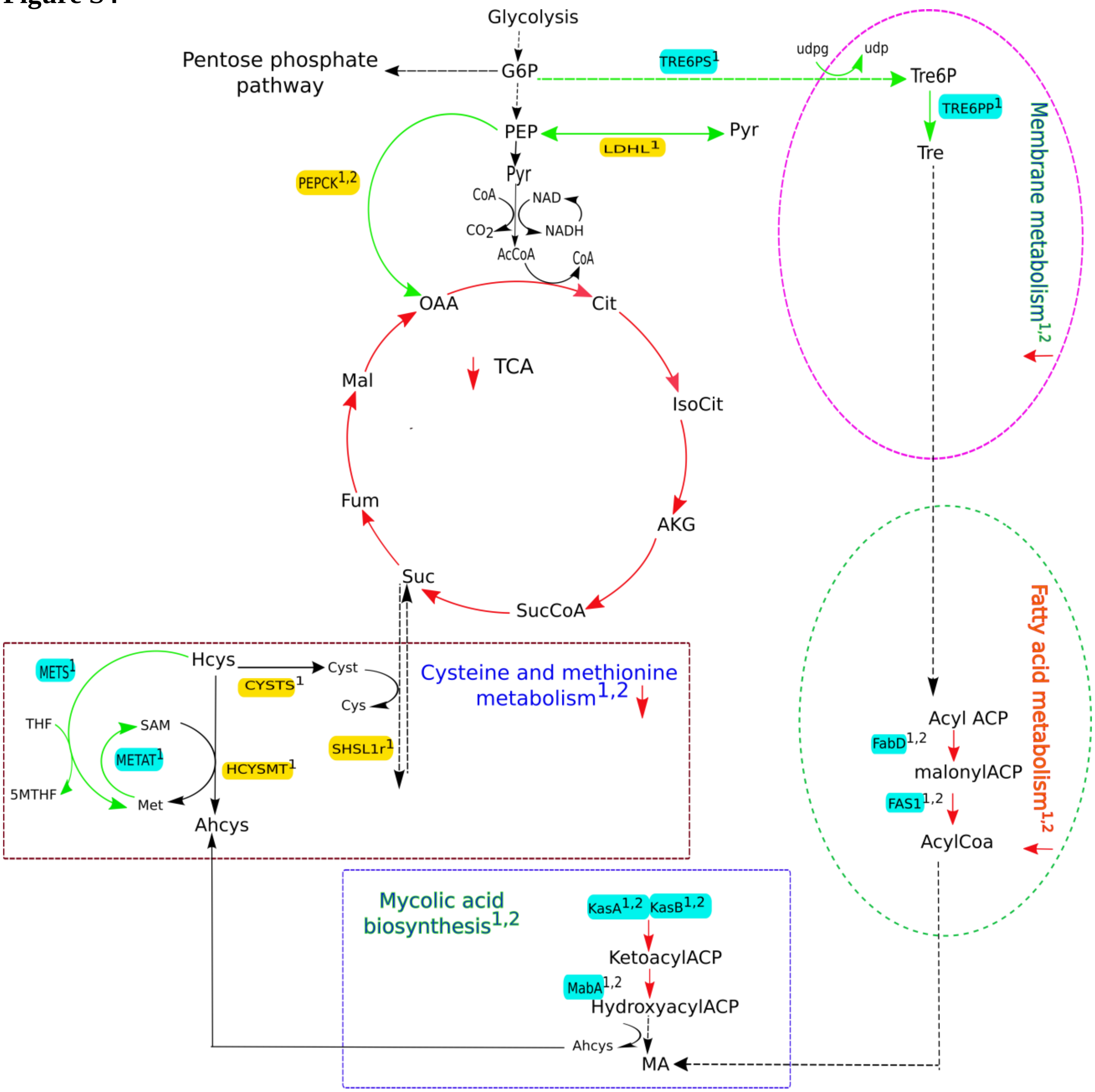

Figure S4. *In-silico* simulation of *M.tb* GEM for nutrient starvation. Red and green arrows indicate a decrease and increase in flux through the reaction respectively when compared to control;1 and 2 in superscript indicate the findings in FBA-M1 and FBA-M2 respectively. TCA, mycolic acid synthesis and fatty acid synthesis pathway showed maximal down-regulation during nutrient starvation. Oxaloacetate (OAA) synthesis via phosphoenolpyruvate carboxykinase (PEPCK)becomes active under nutrient starvation, suggesting a role of this anaplerotic reaction during in vivo survival of *M.tb*. Refer Figure 4 in the main text.

Figure S5

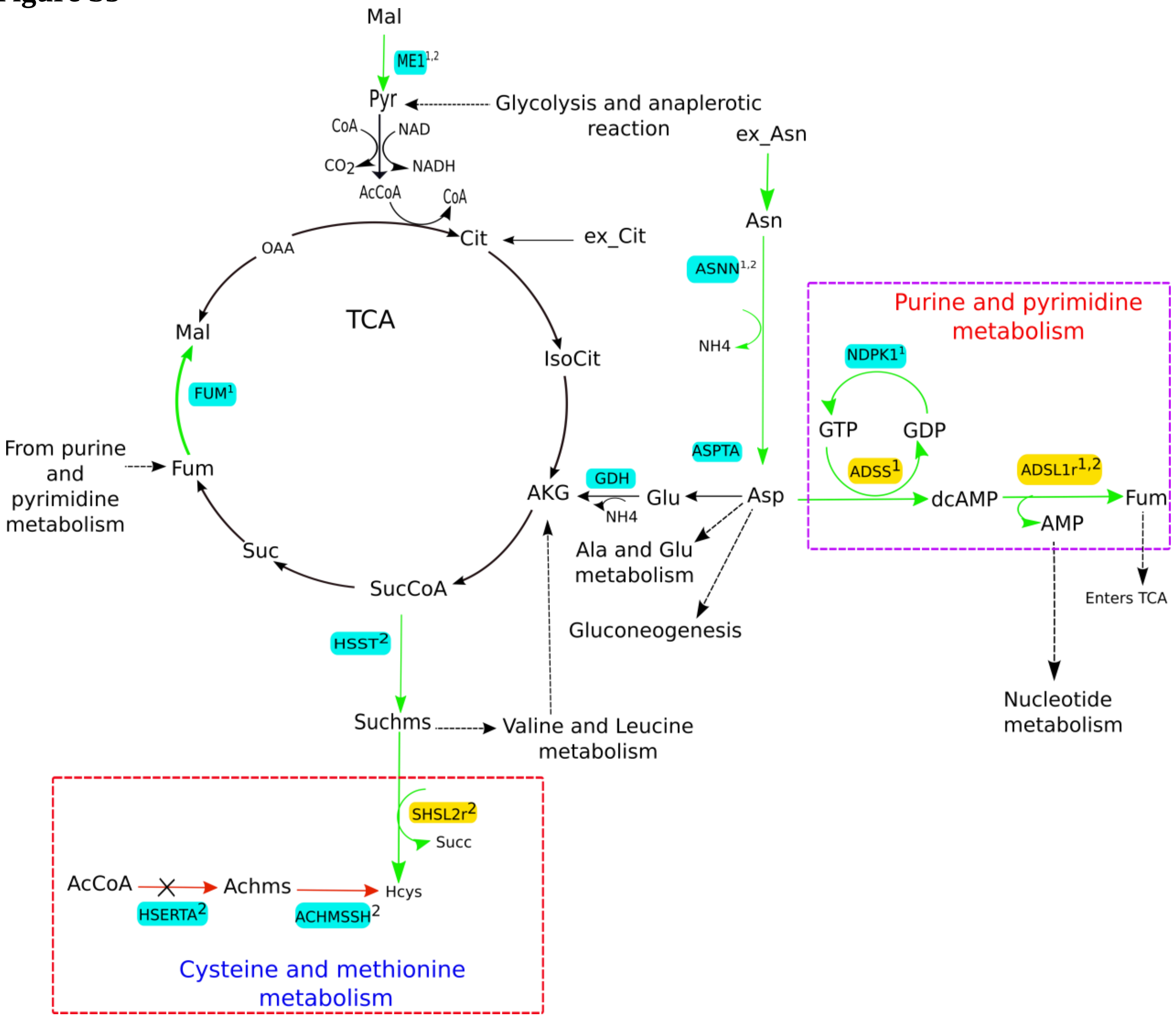

Figure S5. *In-silico* simulation of *M.tb* GEM for acid stress.
Red and green arrows indicate a decrease and increase in flux through the reaction respectively when compared to control. Reactions with no significant difference in flux compared to control are shown in black arrows. ex_ represent external metabolites. FBA results showed significantly increased flux (compared to control and other stress conditions) in the conversion of asparagine (Asn) to aspartate (Asp) through L-asparaginase (ASNN) thereby releasing ammonia. Asp so formed showed higher utilization in the purine and pyrimidine metabolism and released more fumarate via adenylosuccinate synthase (ADSS) and adenylsuccinate lyase (ADSL1r), which consequently enters into the TCA cycle to participate in fumarase (FUM) mediated reaction to produce malate (Mal). Homoserine O-trans-acetylase (HSERTA) and O-acetyl homoserine sulfhydrylase (ACHMSSH) mediated reactions carried zero flux under acid stress unlike control. 1 and 2 in superscript indicate the findings in FBA-M1 and FBA-M2 respectively. ME1, malic enzyme; HSST, homoserine O-succinyltransferase; SHSL2r, O-succinyl homoserine lyase; GDH, glutamate dehydrogenase; ASPTA, aspartate transaminase. Refer Figure 4 in the main text.

Figure S6

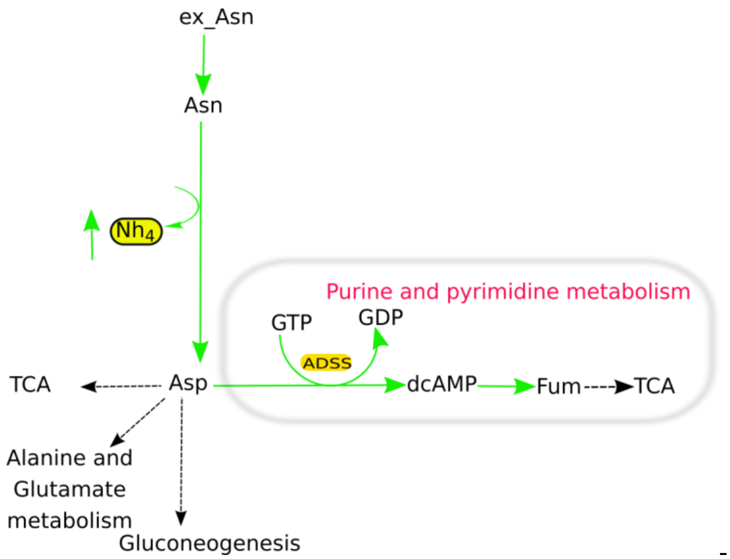

Figure S6. Increased ammonia release under acid stress.
Under acid stress conditions higher aspergine (Asn) uptake was observed that formed aspartate (Asp) releasing increased amount of ammonia. The Asp so formed could have many possible routes of consumption, but the study showed higher utilization of Asp via adenylosuccinate synthase (ADSS) in purine and pyrimidine metabolism. Green arrows indicate higher flux thorugh respective reactions under acid stress. ex_ indicate external metabolite. Refer Figure 4 in the main text.

Figure S7

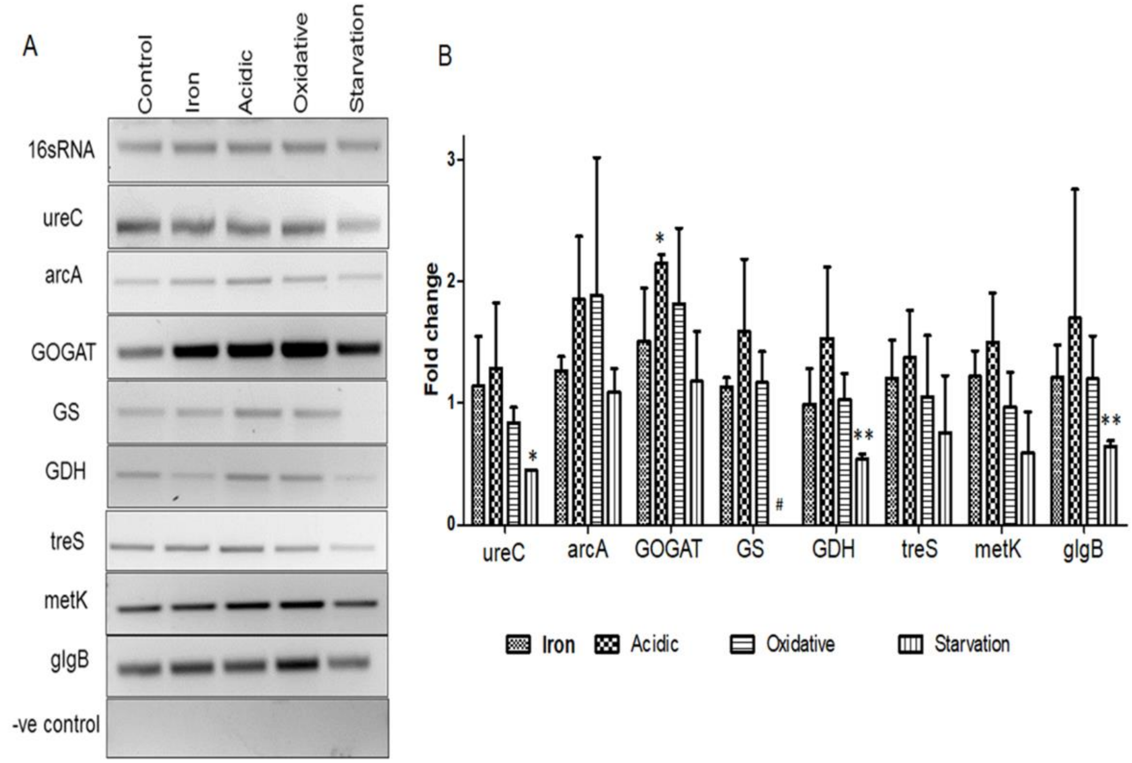

Figure S7: Expression profiling using semi-quantitative RT-PCR of some selected genes. (A) Representative gel picture of semi-quantitative RT-PCR of GS, GDH, GOGAT, ureC, arcA, treS, metK, and glgB by RT-PCR during the microbicidal stresses (viz., acid stress, oxidative stress, iron deprivation, and nutrient starvation) with 16sRNA as a loading control. Lane corresponding to –ve control confirms that there was no genomic DNA contamination. [GS- glutamate synthetase; GDH- glutamate dehydrogenase; GOGAT- glutamine oxoglutarate aminotransferase; ureC- urease C; arC- arginine deaminase; treS- trehalase synthase; metK- methionine adenosyltransferase; glgB- 1,4-alpha-glucan branching enzyme. (B) Densitometric analysis of semi-quantitative RT-PCR of two biological replicates. PCR products of each stress condition were normalized to its respective 16s rRNA. Fold change as compared to the transcript levels of control was calculated. The error bars represent ± standard deviation. ** indicated p value < 0.005 and * indicated p value < 0.05 as compared to control. '#' indicates below detectable range.

Figure S8

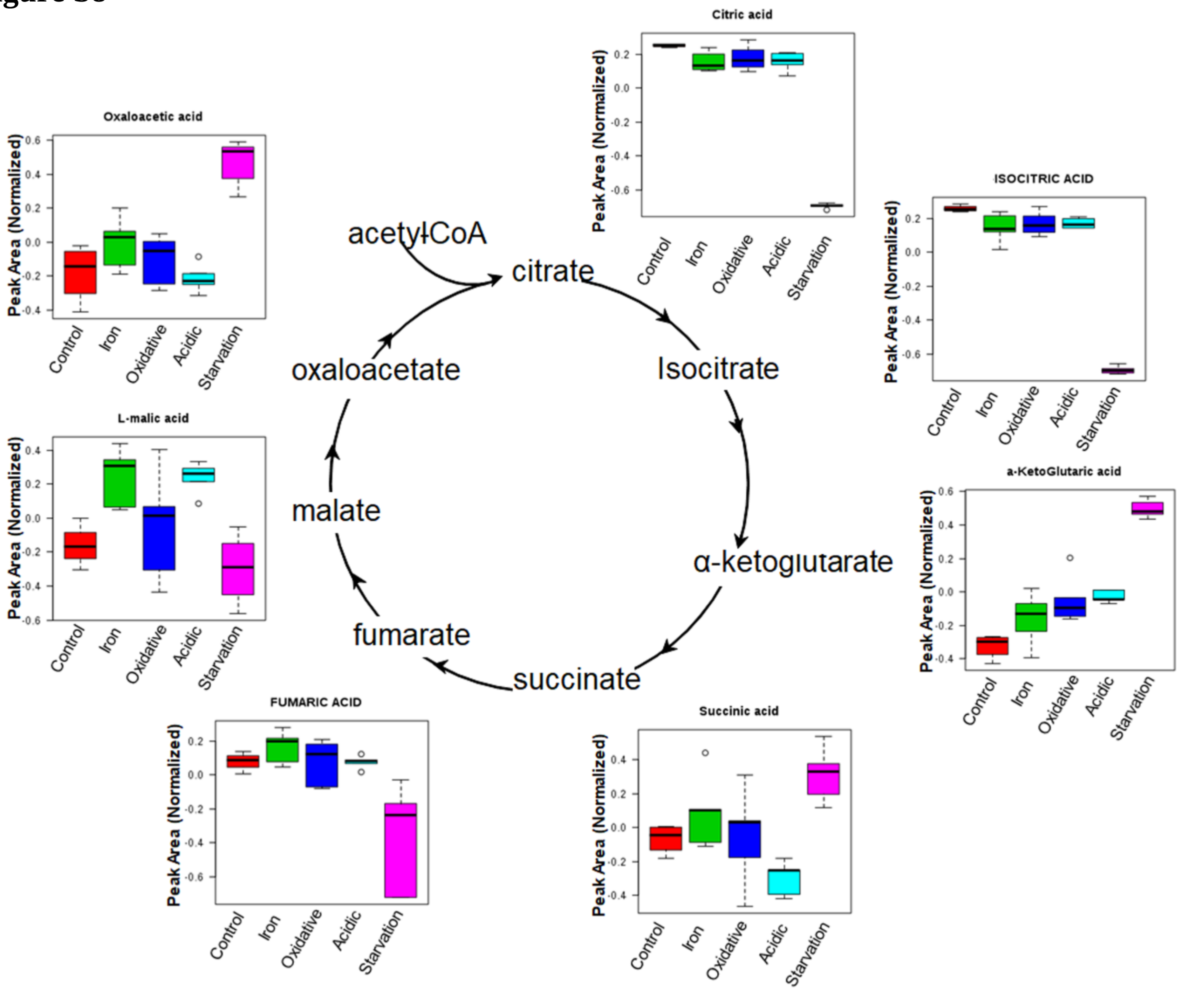

Figure S8: Alterations in TCA cycle of *M.tb* during adaptation to the stresses. Schematic representation of the TCA cycle along with the levels of measured intermediary metabolites of the pathway during each of the stresses.

Figure S9

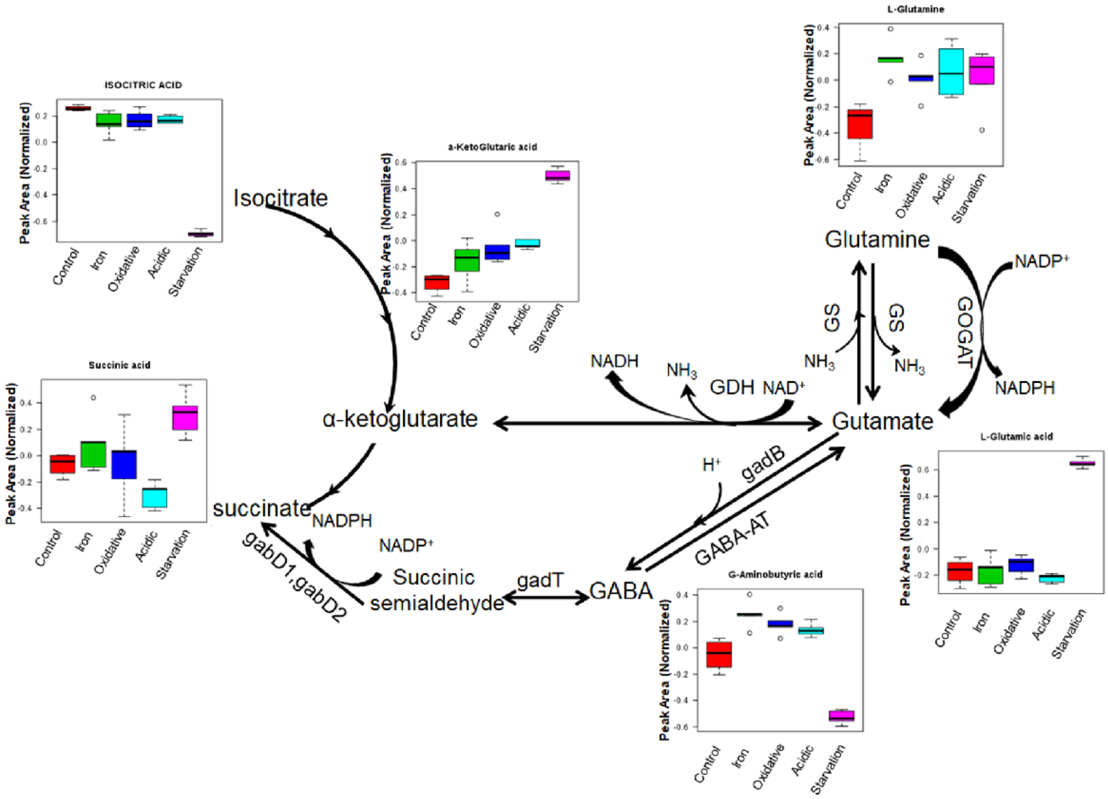

Figure S9: Anaplerotic reactions in the TCA cycle and GABA shunt pathways in *M.tb*. TCA cycle is showing anaplerosis node where glutamate is converted to α-ketoglutarate resulting in the release of ammonia and NADH that possibly contribute towards adaptation to acidic and oxidative stress respectively. GS- glutamate synthetase; GDH- glutamate dehydrogenase; GOGAT- glutamine oxoglutarate aminotransferase; gadB - glutamate decarboxylase; GABA-aminotransferase-GABA-AT; gabD1 and gabD2- succinate-semi aldehyde dehydrogenase.
